# Leveraging edge-centric networks complements existing network-level inference for functional connectomes

**DOI:** 10.1016/j.neuroimage.2022.119742

**Published:** 2022-11-08

**Authors:** Raimundo X. Rodriguez, Stephanie Noble, Link Tejavibulya, Dustin Scheinost

**Affiliations:** a Interdepartmental Neuroscience Program, Yale School of Medicine, 333 Cedar Street, New Haven, CT 06510, USA; b Department of Radiology and Biomedical Imaging, Yale School of Medicine, 330 Cedar Street, New Haven, CT 06520, USA; c Department of Biomedical Engineering, Yale School of Engineering and Applied Science, 17 Hillhouse Avenue, New Haven, CT 06511, USA; d Department of Statistics and Data Science, Yale University, 24 Hillhouse Avenue, New Haven, CT 06511, USA; e Child Study Center, Yale School of Medicine, 230 South Frontage Road, New Haven, CT 06519, USA; f Wu Tsai Institute, Yale University, 100 College Street, New Haven, CT 06510, USA

**Keywords:** Functional connectivity, Network-level statistics, Power, Edge-centric networks

## Abstract

The human connectome is modular with distinct brain regions clustering together to form large-scale communities, or networks. This concept has recently been leveraged in novel inferencing procedures by averaging the edge-level statistics within networks to induce more powerful inferencing at the network level. However, these networks are constructed based on the similarity between pairs of nodes. Emerging work has described novel edge-centric networks, which instead use the similarity between pairs of edges to construct networks. In this work, we use these edge-centric networks in a network-level inferencing procedure and compare this novel method to traditional inferential procedures and the network-level procedure using node-centric networks. We use data from the Human Connectome Project, the Healthy Brain Network, and the Philadelphia Neurodevelopmental Cohort and use a resampling technique with various sample sizes (*n*=40, 80, 120) to probe the power and specificity of each method. Across datasets and sample sizes, using the edge-centric networks outperforms using node-centric networks for inference as well as edge-level FDR correction and NBS. Additionally, the edge-centric networks were found to be more consistent in clustering effect sizes of similar values as compared to node-centric networks, although node-centric networks often had a lower average within-network effect size variability. Together, these findings suggest that using edge-centric networks for network-level inference can procure relatively powerful results while remaining similarly accurate to the underlying edge-level effects across networks, complementing previous inferential methods.

## 1. Introduction

Connectomics has brought about novel discoveries that expand upon what can be observed through activation maps (Fornito et al., 2013), providing a method that allows us to study the brain as a complex graph (Bassett and Sporns, 2017). Formally, the brain regions comprise the set of graph nodes, and the temporal similarity, or connectivity, between the brain regions describes the edge weights between nodes. Such a formulation provides a rich source of data for which statistical associations can be inferred between edge weights and subject phenotypes. However, a connectome is necessarily large because the number of edges scales with the square of the number of spatial regions. With this increase in dimensionality arises the need to efficiently correct for multiple comparisons to adequately control false rejections of the null hypothesis without over-restricting the true rejections.

As simple Bonferroni correction is too stringent (Brett et al., 2004), a number of methods have been prescribed for controlling various metrics of false positive detections when using connectomes for statistical inference. One popular method is to control the false discovery rate (FDR), which can manifest in many implementations, such as the Storey method (Storey, 2002). Controlling FDR is much less stringent than prior methods because it only controls the family-wise error rate (FWER) in the weak sense (Benjamini and Hochberg, 1995). Additionally, connectome-specific methods have been developed. For example, the Network-Based Statistic (NBS) (Zalesky et al., 2010), as well as extensions of NBS (Baggio et al., 2018; Vinokur et al., 2015), controls false positive detections by leveraging the network structure of the brain. Briefly, this is achieved by employing a similar technique as cluster-based corrections used for statistical parametric mapping (SPM) (Friston et al., 1996; Nichols and Hayasaka, 2003), but instead of considering clusters to be a set of spatially contiguous suprathreshold voxels, clusters are defined as topologically contiguous suprathreshold edges (*i.e.*, connected components). NBS has been demonstrated to increase statistical power compared to previous methods in situations where phenotype-driven connectivity delineates a set of connected components (Zalesky et al., 2010).

Although NBS and its extensions have offered great strides in improving statistical inference on the human connectome, one shortcoming is that they do not capitalize on the explicit network structure of the connectome. However, as the field of network neuroscience has developed, the classification of networks (e.g., the default mode network) has become a prominent topic of interest (Uddin et al., 2019), and these networks have been demonstrated to capture important phenotypic relationships such as in the cases of sex (Allen et al., 2011; Biswal et al., 2010; Bluhm et al., 2008; Filippi et al., 2013; Tomasi and Volkow, 2012) and metrics of intelligence (Dubois et al., 2018), as well as the interaction between the two (Jiang et al., 2020). An attempt to utilize the large-scale network structure of functional connectomes yielded the Constrained Network-Based Statistic (cNBS) which both leverages and preserves the uniqueness of large-scale networks to control false detections (Noble, 2022; Noble and Scheinost, 2020). cNBS accomplishes this by using the mean within-network edge value to operate on the network level. It was found to perform particularly well compared to NBS in the presence of smaller-than-medium effect sizes, suggesting that the method one should use to correct for multiple comparisons may depend on the operating regime of effect size magnitudes.

However, it should be noted that the networks used in cNBS are derived from pairwise *node* similarity graphs (*i.e.,* functional connectomes), even though the units for which a given hypothesis is tested are edges. Specifically, if the network partition consists of *N* predefined node-centric networks, this yields *N* clusters of edges that belong completely within a network and *N(N−1)/2* clusters of edges that belong between two networks for a total of *N(N*+*1)/2* clusters of edges. This is an important point because recent studies have sought to study pairwise edge relationships, and other higher-order relationships, rather than the traditional pairwise node relationships (Chumin et al., 2022; Esfahlani et al., 2021; Faskowitz et al., 2022, 2021, 2020; Gao et al., 2020; Jo et al., 2021a, 2021b; Novelli and Razi, 2022; Owen et al., 2021; Sporns et al., 2021). Furthermore, it has been demonstrated that although edge-centric networks recapitulate many of the characteristics of node-centric networks, there are distinct differences between the two types of networks (Jo et al., 2021b).

Therefore, in this work, we introduce a novel implementation of cNBS—named edge-centric cNBS (ecNBS)—that uses edge-centric networks (derived from the similarity between pairs of edges) to constrain the multiple comparison correction. ecNBS is motivated by the logic that edgewise functional connectivity would construct a more useful set of networks than nodewise functional connectivity. In addition, edge-centric networks afford the advantage to directly test the relevance of the number of networks on cNBS performance, whereas clustering edges from node-centric networks restricts the numbers of networks that can be used in cNBS. To construct the edge-centric networks, we use a method similar to one that has been used to construct edge hyper-graphs (Gu et al., 2017) in conjunction with the Normalized Cut algorithm previously used to define parcellations in large graphs (Shen et al., 2013, 2010). Notably, we do not employ the method implemented in Faskowitz et al., 2020 to define pairwise edge similarity, but rather elect to use a different method which is better suited for our purposes. We first evaluate the ability of ecNBS to control both FWER and FDR with varying sample sizes, varying numbers of edge-centric networks, and varying ground truth effect size distributions using a previously-defined benchmarking procedure (Cremers et al., 2017; Geuter et al., 2018; Noble, 2022; Noble and Scheinost, 2020). We then compare ecNBS to the existing methods to demonstrate in which parameter spaces our method offers improvements in power over previous methods and in which spaces our method falls short. Next, we explore how well edge-centric and node-centric networks capture the effects contained in their constituent edges, hypothesizing that edge-centric networks will outperform node-centric networks in this regard. Lastly, we show the results one could expect from each method when scanning for sex differences in a large dataset.

## 2. Materials and methods

### 2.1. Datasets

Three datasets were used in our benchmarking procedure to evaluate the performance of all inferential methods tested here–the Human Connectome Project (HCP) (Van Essen et al., 2013), the Philadelphia Neurodevelopmental Cohort (PNC) (Satterthwaite et al., 2016, 2014), and the Healthy Brain Network (HBN) (Alexander et al., 2017). The HCP dataset is composed of individuals of ages between 22 and 35 years from the United States. The PNC dataset consists of individuals between the ages of 8 and 23 years from the greater Philadelphia area. The HBN dataset has scans from individuals between 5 and 22 years old. A fourth dataset collected at Yale, the Yale-High Resolution Controls (YHRC) dataset, was used to construct edge-centric networks and consists of 94 subjects.

### 2.2. Processing

For the HCP S1200 release (Van Essen et al., 2013), we used the resting-state data collected on day 1 which consisted of 1200 individual time points per run. Data with a maximum frame-to-frame displacement of 0.15 mm or greater were excluded, and we used 1058 subjects that met this criteria (males: 489, females: 569). For the PNC dataset (Satterthwaite et al., 2016, 2014), all resting-state scans are 6 minutes long. After excluding subjects for missing scans/data and excessive motion (> 0.25 mm), 766 subjects remained (right-handed individuals: 660, left-handed individuals: 106). For the HBN dataset (Alexander et al., 2017), all resting-state scans are 10 minutes long. After excluding subjects for missing scans/data and excessive motion (> 0.25 mm), 817 subjects remained (right-handed individuals: 713, left-handed individuals: 104). For the YHRC dataset, all resting-state scans are 5.55 minutes, of which there are 6 scans per individual. After excluding subjects for missing scans/data and excessive motion (> 0.15 mm), 94 subjects remained. Note that the PNC and HBN datasets had a more permissive motion threshold because they are developmental samples and many subjects have at least one mental health diagnosis.

For the HCP, we started with the minimally preprocessed data (Glasser et al., 2013). For the PNC, HBN, and YHRC datasets, structural scans were first skull stripped using an optimized version of the FMRIB’s Software Library (FSL) (Smith et al., 2004) pipeline (Lutkenhoff et al., 2014) and registered into common space. Functional images were motion corrected using SPM. For all datasets, further preprocessing steps were performed using BioImage Suite (Joshi et al., 2011). These included regressing 24 motion parameters, the mean white matter, CSF, and gray matter time series, removing the linear and quadratic trends, and low-pass filtering (Gaussian filter with cutoff frequency ~0.12 Hz). After preprocessing, connectomes were constructed using the Shen268 (268 nodes) atlas (Shen et al., 2013). This involved computation of the mean time courses for each of the 268 nodes (i.e., averaging the time courses of all constituent voxels). For a supplemental analysis (SI Fig. 7), the HCP and YHRC data were also processed with the Shen368 (368 nodes) atlas to generate data for benchmarking and network construction, respectively. During this, 4 HCP subjects and 7 YHRC subjects failed to be processed and were removed from the supplemental analysis. For all datasets, connectomes were generated by calculating the Pearson’s correlation between each pair of the mean time series and then taking the Fisher transform of these correlations. For datasets with more than one resting-state run, connectomes were averaged together.

### 2.3. Phenotypes of interest

Sex and fluid intelligence (IQ) are some of the most frequently studied phenotypes and were thus used for evaluation here. We studied these phenotypes in the HCP dataset. Additionally, it has recently been shown that handedness exhibits effect sizes similar to those of sex (Tejavibulya et al., 2022), and as increased interest in handedness is emerging, we included it in our evaluations. Handedness was studied in two ways: as a binary variable in the PNC dataset and as a continuous variable (left-dominant to right-dominant scale from −100 to 100) in the HBN dataset. Finally, since age is also a frequent phenotype of interest, we studied this phenotype in the PNC dataset.

### 2.4. Edge-centric constrained network-based statistic (ecNBS)

**Fig. 1.**
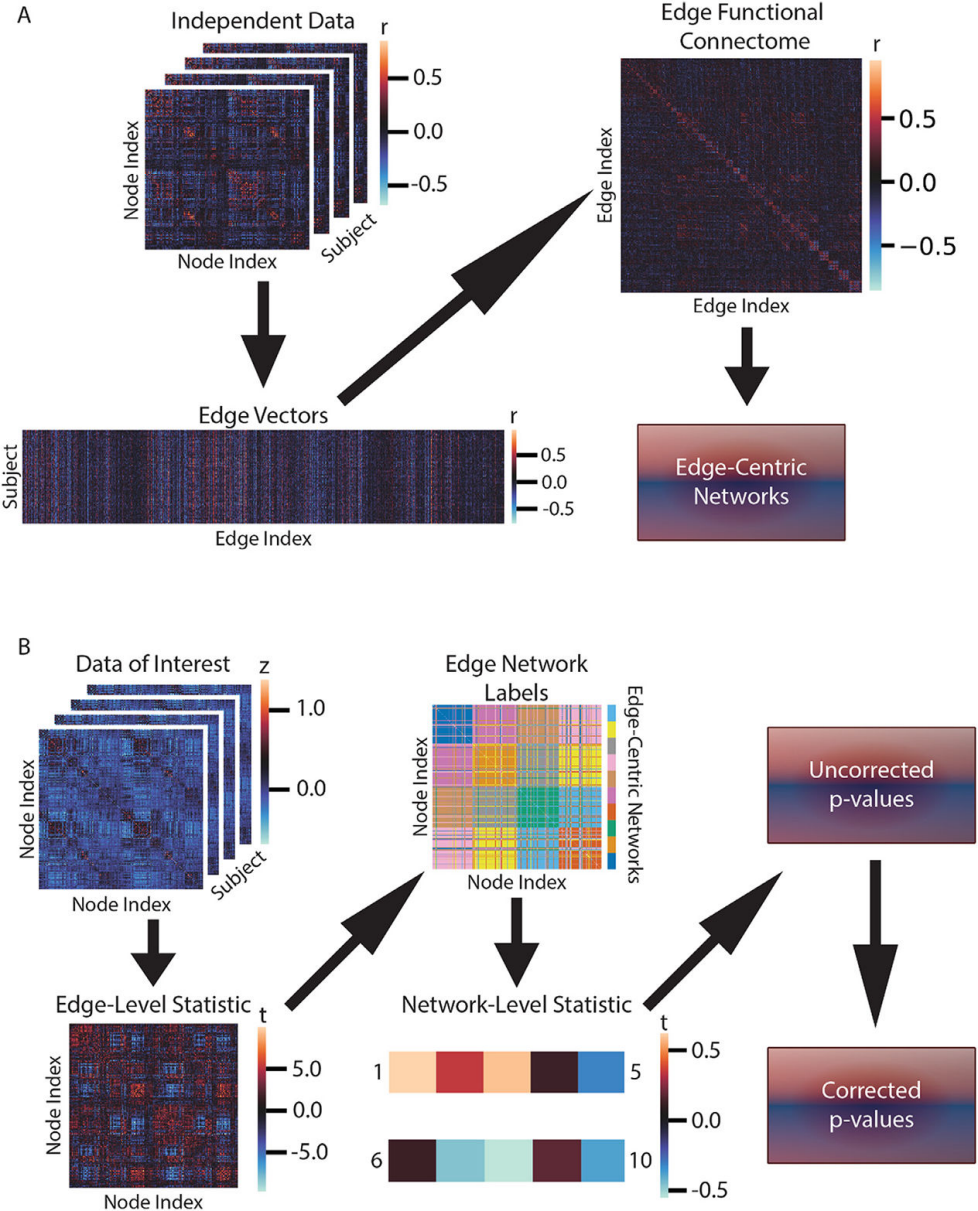
Method overview. Depicted are A) the method for edge-centric network construction and B) the inferential procedure for ecNBS. A) Edge-centric network construction begins with a set of independent connectomes. For each edge, values are concatenated across subjects to obtain a matrix of edge vectors. Pair-wise correlations of the columns of this matrix are computed to construct the edge functional connectome (shown are the first 1000 edges for visualization). Finally, the Ncut algorithm is applied to obtain the desired number of edge-centric networks. B) To perform ecNBS, one begins with a set of connectomes on which a statistical test is applied to obtain the edge-level statistics. The previously constructed edge-centric networks are then used to assign each statistic value to a group. Statistic values are then averaged within-group to obtain the network-level statistics (shown here is a fabricated example of 10 networks for visualization). Permutation testing as described in Section 2.4 is performed to obtain an uncorrected *p*-value for the network-level statistics. Finally, FDR correction is applied to obtain the corrected, network-level *p*-values.

The method of primary interest in this work is our proposed edge-centric constrained network-based statistic (ecNBS), which is similar to a recently developed method known as the constrained network-based statistic (cNBS) (Noble, 2022; Noble and Scheinost, 2020). This method consists of two phases: constructing the edge-centric networks and running the inferential procedure. A visual interpretation of the method is depicted in Fig. 1, and a visualization of one edge-centric network partition is shown in SI Figs. 14-17.

#### 2.4.1. Edge-centric network construction

Using the YHRC dataset, connectomes were inverse Fisher transformed and used to construct edge vectors. Briefly, an edge vector was created by concatenating the value of the edge across subjects. Once this was performed for all edges, an edge connectome was created by correlating all pairs of edge vectors to create a group-level 35,778 × 35,778 matrix (Gu et al., 2017). Hence, each entry in this matrix measures how similar two edges are across a population, and the matrix can be used to construct networks assumed to be consistent across individuals. The edge connectome was then input to the Normalized Cut algorithm. In this algorithm, the correlation values are first converted to distance, ranging from 0 to 4, and then weighted by a Gaussian function for which the mean was set to 0 and the standard deviation to $\frac{0.25}{\sqrt{2}}$. Then for each number of networks (*n*= 35, 55, 75, 95, 115, and 135), the first *n* non-trivial eigenvectors of the normalized Laplacian matrix were computed. These eigenvectors were then used to partition the graph into *n* clusters as described in (Yu and Xi, 2003). In this way, we obtained six distinct network partitions with varying numbers of edge-centric networks. For a supplemental analysis (SI Fig. 6), another six network partitions were identified using k-means instead of Normalized Cut.

#### 2.4.2. Inferential procedure

First, the statistic of interest is performed at the edge-level across the entire connectome. The edge-level test statistics are then averaged within each edge-centric network to obtain the network-level statistic. For each network, a null distribution of network statistics is constructed by permuting the phenotype label, as detailed in the seminal work on permutation testing (Nichols and Holmes, 2002), and re-computing the network-level statistic using 1000 permutations. Next, the uncorrected *p*-value for each network is obtained by determining in which percentile of the network-specific null distribution the true statistic resides. Finally, the Simes procedure to control false discovery rate (FDR) (Simes, 1986) is used to correct the *p*-value for each network.

### 2.5. Inferential methods for comparison

#### 2.5.1. Edge-level + FDR

The first inferential method used for comparison here was the Storey method for FDR, which controls the expected proportion of false positives to total positives (Benjamini and Hochberg, 1995; Storey, 2002). Throughout this work, we simply refer to this method as edge-level + FDR. To implement this method, we used mafdr.m from Matlab.

#### 2.5.2. NBS

The next method used was the network-based statistic (NBS) (Zalesky et al., 2010). Briefly, this method takes the test statistic (*t* value) for each edge in the connectivity matrix and binarizes according to a threshold, here set to t=3.1. Since the connectivity matrix is equivalent to a graph, the resulting binary matrix describes an unweighted graph; the graph’s connected components (if there are any) are identified, and their sizes are recorded. The phenotypic measures for each subject are then permuted, and the test statistics are recalculated. For each permutation–1000 were used here–the size of the largest connected component is recorded and used to define a null distribution of maximum cluster sizes. Once the distribution is constructed, the familywise error rate (FWER)-corrected *p*-value for each connected component in the experimental data is calculated by determining in which percentile of the null distribution its size resides and subtracting that percentile from 1. Setting the FWER-corrected *p*-value threshold to 0.05 controls FWER to be less than 5% in the weak sense because in no more than 5% of permutations, in which the null hypothesis is assumed to be true everywhere, was a connected component of greater size found. This method is analogous to previous cluster-based permutation methods (Friston et al., 1996; Nichols and Hayasaka, 2003) except that proximity is defined topologically rather than geometrically.

#### 2.5.3. cNBS

A recently developed method known as the constrained network-based statistic (cNBS) (Noble, 2022; Noble and Scheinost, 2020) was also evaluated. Since ecNBS follows the same procedure as cNBS, we refer the reader to Section 2.4. The only difference between the two methods was the network partition used. cNBS used ten node-centric networks (Finn et al., 2015; Noble et al., 2017), constructed by using the same Normalized Cut algorithm and YHRC dataset that was used to create the edge-centric networks. These 10 networks partition the nodes, so to partition the edges for the analysis, the pairs of node-centric networks that each edge participates in define the edge partition. Specifically, these networks lead to 10 within network (e.g., DMN to DMN edges) and 45 between network (e.g., DMN to FPN edges) groupings of edges. Importantly, cNBS is generalizable to other predefined network partitions.

### 2.6. Benchmarking

The benchmarking procedure followed the process as in (Cremers et al., 2017; Geuter et al., 2018; Noble, 2022; Noble and Scheinost, 2020). The procedure was performed for each phenotype of interest (see Section 2.3) in the mentioned datasets. First, a random subsample was taken from each of the full datasets; the subsample size was either 40, 80, or 120. Notably, because there were many more right-handed than left-handed subjects in the PNC dataset, the random subsamples for this dataset were deliberately constrained to reflect the full cohort proportion of right-handed individuals to left-handed individuals. Then, for each phenotype, two test statistics were computed for all edges. For HCP sex and PNC handedness, the test statistic for an edge was the *t*-value from the one-tailed t-test for the hypothesis that the edge in males is greater than the edge in females (and vice versa) and the hypothesis that the edge in right-handed subjects is greater than the edge in left-handed subjects (and vice versa), respectively. For HCP IQ, HBN handedness, and PNC age, the test statistics for an edge were the statistic corresponding to the hypothesis that the edge positively correlates with the phenotype and the statistic corresponding to the hypothesis that the edge negatively correlates with the phenotype. Next, each test statistic matrix was input to each of the inferential methods. Thus, for each hypothesis (two per phenotype), the corrected *p*-values were returned according to each method. For edge-level + FDR and NBS, the corrected *p*-values were returned at the edge level. For the cNBS and ecNBS, the corrected *p*-values were returned at the network level. Finally, the corrected *p*-values were thresholded at a level of 0.05 to determine for which edges or networks the null hypothesis was rejected. This process was repeated 500 times for each subsample size.

### 2.7. Performance metrics

#### 2.7.1. Analysis overview

Here, we compared the performance of ecNBS against cNBS using an empirical benchmarking approach and investigated the factors that contribute to ecNBS performance. Factors of interest were the number of edge-centric networks input into the ecNBS algorithm, the sample size of the data, and the ground truth distribution of edge effect sizes. To indirectly control the effect size distributions, a variety of phenotypes and datasets were used in the benchmarking procedure. Hence, we performed benchmarking (see Section 2.6) with 500 random subsamples (repetitions) for the HCP dataset using sex and IQ as the phenotypes, for the HBN dataset using handedness as the phenotype, and for the PNC dataset using handedness and age as the phenotypes. Benchmarking was then performed with different numbers of edge-centric networks and subsample sizes. To compare to existing methods, we also performed benchmarking with the same parameters (except for varying the number of edge-centric networks) using cNBS, NBS, and edge-level + FDR methods.

The main metrics by which to measure performance were true and false detections, power, and a number of false positive rate (FPR) measures–FDR and strong and weak FWER. All metrics were computed at the appropriate level of analysis (either edge, cluster, or network level) depending on the method used in the benchmarking procedure except for power in NBS, which was calculated at the edge level. In all cases, a ground truth was required to determine whether a rejection of the null hypothesis was a true or false detection. Additionally, power was computed as a function of effect size magnitude to compare the inferential methods at each effect size rather than across an entire dataset.

#### 2.7.2. Ground truth

The ground truth effect size matrix was calculated for each phenotype separately and was computed at either the edge or network level. For HCP sex and PNC handedness, the effect size for an edge was calculated as Cohen’s d. For HCP IQ, HBN handedness, and PNC age, the effect size for each edge was calculated as the Cohen’s d equivalent for the Pearson’s correlation between that edge with the phenotype measure, setting Δ = 2*s* where *s* is the sample standard deviation of the phenotype (Mathur and VanderWeele, 2020). To calculate the ground truth effect size for a network, edge values within that network were averaged for each subject, and then the appropriate effect size calculation was performed.

#### 2.7.3. True and false detection

For either of the two hypotheses, an edge, cluster, or network could be either detected or not for a given benchmarking repetition. Notably, if a detection occurred for one hypothesis, it cannot occur in the same component for the other hypothesis in the same repetition. For edge-level + FDR, detections were always computed at the edge level, and for ecNBS and cNBS, detections were always computed at the network level. Thus for the edge and network levels, if the hypothesis for which a component was detected corresponded to the appropriate ground truth effect size sign, it was labeled as a true detection. On the other hand, if the hypothesis for which a component was detected corresponded to the incorrect ground truth effect size sign, it was labeled as a true detection. However, for NBS, detections could either be computed at the edge or cluster level. To compute at the edge level, all edges within a detected cluster underwent the above classification scheme. For a detected cluster to be classified as a true detection, only one edge within the cluster had to be classified as a true detection. If no edge within the cluster could be classified as a true detection, the cluster was labeled as a false detection.

#### 2.7.4. Power

Power is defined as the expected probability a null hypothesis will be correctly rejected. For edge-level + FDR, power was computed at the edge level, and for ecNBS and cNBS, power was computed at the network level. For NBS in this case, because the spatial extent of clusters could vary across repetitions, power was computed at the edge level. Thus, for an edge or a network, the power was the number of true detections for that component divided by the number of repetitions.

Power was also estimated for a component of a particular effect size magnitude. To do so, for a given inferential method, the appropriate ground truth effect sizes were pooled across all four datasets. The absolute value was taken and they were sorted; the corresponding power measurements were also sorted according to the new effect size ordering. Then, overlapping windows (75%) of width 0.1 (corresponding to Cohen’s d) were applied to the effect size vector, averaging the values within the window as well as the corresponding power values. In this way, we were able to obtain a smoothed function of effect size magnitude versus power.

#### 2.7.5. FDR

FDR is defined as the expected proportion of false detections to total detections when hypotheses are tested for multiple components. FDR for all inferential methods was calculated at the level of inference: edge for edge-level + FDR, cluster for NBS, and network for ecNBS and cNBS. Thus, for a single benchmarking repetition, the FDR was the number of falsely-detected components divided by the total number of detected components, and the average was taken across repetitions to obtain its expected value. Importantly, there could be repetitions for which an inferential method incurred no detections in which case the FDR in that repetition would be undefined. To avoid this scenario’s potential to interfere with obtaining an expected value, the FDR for these repetitions were set to zero, as prescribed by (Benjamini and Hochberg, 1995). With FDR, the objective was to be below 5%.

#### 2.7.6. Strong FWER

FWER in the strong sense is defined as the expected probability that the number of falsely detected components is greater than zero when the null hypothesis is known to be false for some components. Strong FWER for all inferential methods was calculated at the level of inference: edge for edge-level + FDR, cluster for NBS, and network for ecNBS and cNBS. Thus, strong FWER was computed as the number of repetitions for which the number of falsely detected components was at least one divided by the total number of repetitions. For FWER, the objective was to be below 5%; since we repeated the benchmarking procedure 500 times, the expected 95% confidence interval for FWER was 5% +/− 2% (Wilson, 1927; Winkler et al., 2014; cf. Eklund et al., 2016), so the maximum allowed mean FWER for each method was 7%.

#### 2.7.7. Weak FWER

FWER in the weak sense is defined as the expected probability that the number of falsely detected components is greater than zero when the null hypothesis is known to be true for all components. This first required a slight modification to the benchmarking procedure which was run again except that in each repetition, the subject indices were randomly permuted to disrupt the underlying phenotypic differences. For HCP sex and PNC handedness, the test statistic for an edge was the *t*-value from the one-tailed t-test for the hypothesis that the edge was greater in group 1 than in group 2. For HCP IQ, HBN handedness, and PNC age, the test statistic for an edge was the statistic corresponding to the hypothesis that the edge positively correlated with the phenotype. Benchmarking then proceeded as explained in Section 2.6, and detected components were categorized as either true or false detections as described above. Weak FWER for all inferential methods was calculated at the level of inference: edge for edge-level + FDR, cluster for NBS, and network for ecNBS and cNBS. Thus, weak FWER was computed as the number of repetitions for which the number of falsely detected components was at least one divided by the total number of repetitions. Like strong FWER, control of weak FWER for a threshold of 5% should be within 2%.

### 2.8. Data and code availability statement

The data used in this study for inference and benchmarking are open-source: HCP (https://db.humanconnectome.org), HBN (http://fcon_1000.projects.nitrc.org/indi/cmi_healthy_brain_network/sharing.html), and PNC (https://www.ncbi.nlm.nih.gov/projects/gap/cgi-bin/study.cgi?study_id=phs000607.v3.p2). The Yale data used in this study to construct edge-centric networks are open-source and available here: http://fcon_1000.projects.nitrc.org/indi/retro/yale_hires.html. Code used for node-centric and edge-centric network construction can be found at https://www.nitrc.org/projects/bioimagesuite/. Code used for bench-marking, inference, and summarization/reorganization by atlas is available here: https://github.com/SNeuroble/NBS-benchmarking.

### 2.9. Ethics statement

All human subject data are publicly available and were collected under the guidance of the local IRBs of the data collection sites. Yale Human Research Protection Program (HIC #2000023326) approved secondary analyses of these datasets.

## 3. Results

### 3.1. ecNBS adequately controls false positive rate

**Fig. 2.**
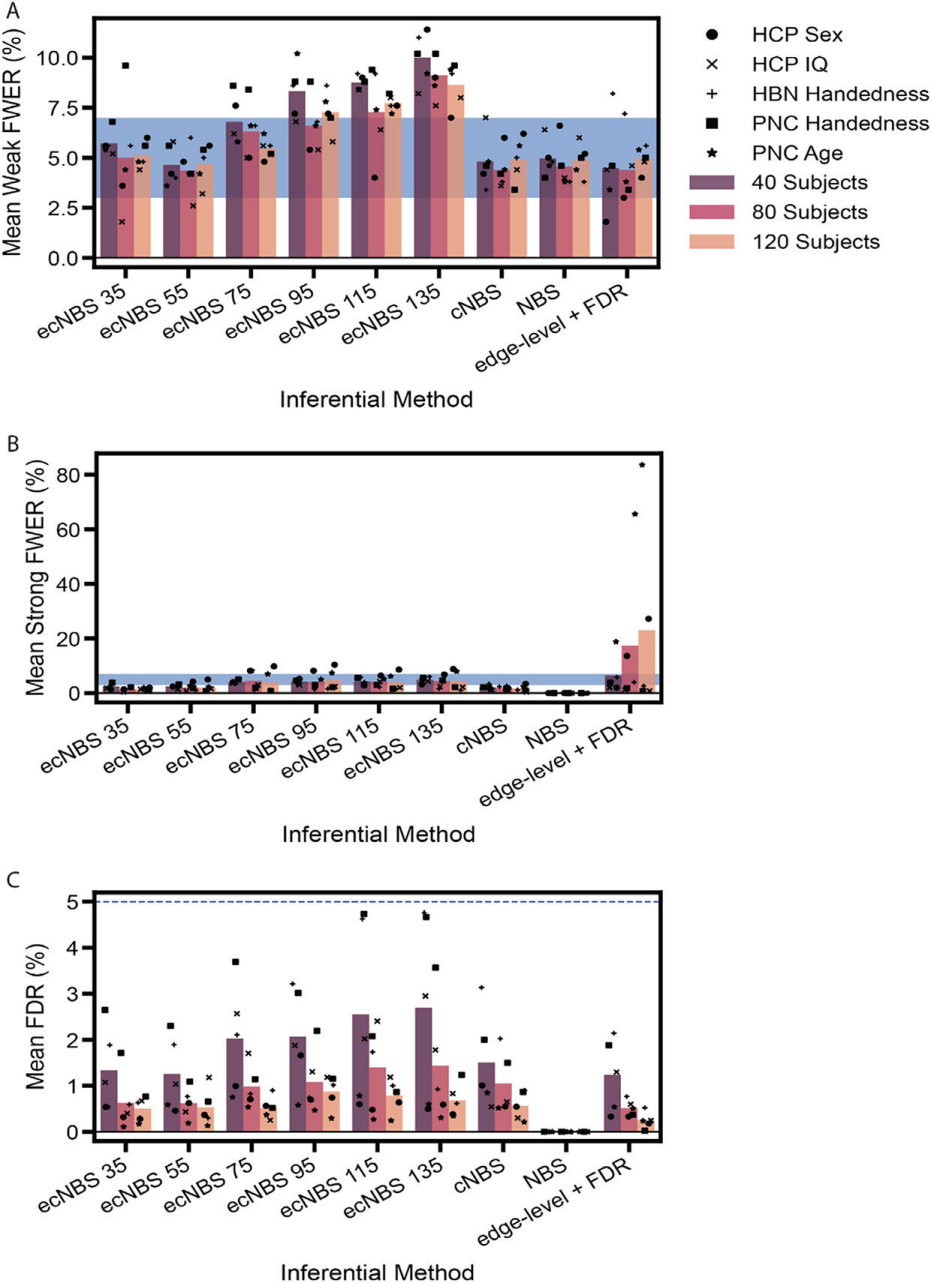
False positive metrics for benchmarking. We used A) weak FWER, B) strong FWER, and C) FDR to assess each inferential method’s ability to control the false positive rate. For every dataset, each metric was computed at the repetition level and then averaged across benchmarking repetitions. Each set of bars on the x-axis represents an inferential method that was used for benchmarking. The colors of the bars correspond to the sample size used during benchmarking. The points correspond to the mean metric for a particular dataset, and the bars correspond to the average across datasets. The shaded blue regions in A) and B) are the 95% confidence interval around a FWER of 5% for 500 benchmarking repetitions, and the blue line in C) is set at 5% to show the permissible FDR.

First, we calculated various metrics of the FPR for all inferential methods. Fig. 2 shows the mean weak FWER, strong FWER, and FDR across datasets for each inferential method and subsample size as well as the mean metrics at the single dataset level. On average, ecNBS controlled strong FWER for all subsample sizes and always controlled FDR. ecNBS also controlled weak FWER on average when fewer numbers of edge-centric networks were used, with the mean weak FWER increasing with increasing numbers of edge-centric networks. cNBS followed a similar pattern to that of ecNBS except that it also always controlled weak FWER on average. ecNBS with 55 edge-centric networks and NBS were the only methods to control all three metrics of false positive rate absolutely. Finally, edge-level + FDR always controlled weak FWER for all but the HBN handedness dataset, always controlled strong FWER for all but the sex and age datasets, and always adequately controlled FDR. In general, all methods controlled the false positive rate. Yet more specifically, the results here indicate that there may be a limit to which each of these methods can control various aspects of false positive detection, which may in turn depend on the effect sizes associated with the given phenotype.

### 3.2. ecNBS performance: Effect of number of edge-centric networks, subsample size, and effect size distribution

**Fig. 3.**
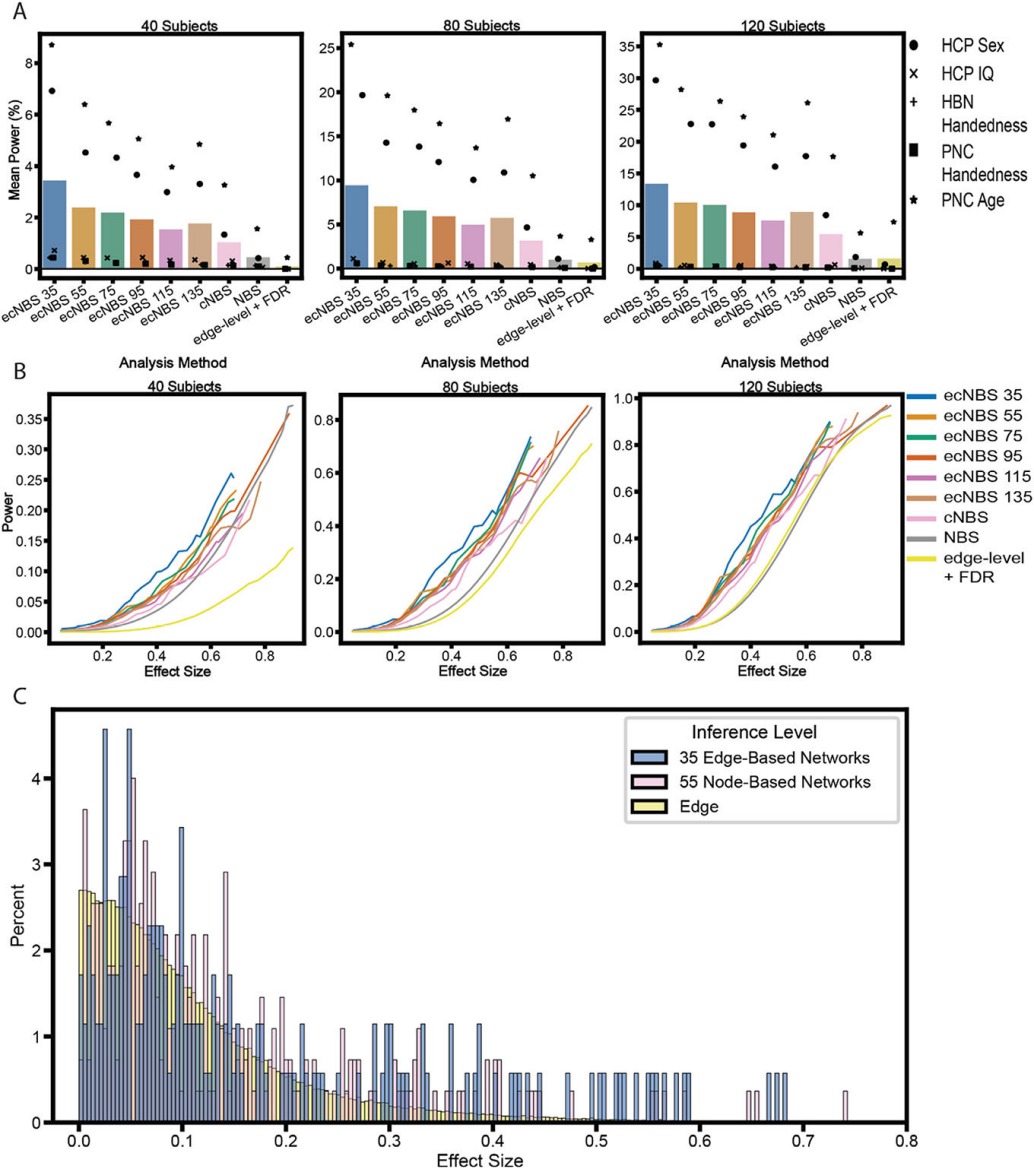
Mean power in relation to ground truth effect size distributions. A) Power is averaged across components (i.e. networks or edges) in a dataset and then averaged across datasets. Positions and colors of the bars on the x-axis correspond to the method used with benchmarking. Points represent the mean power for benchmarking performed on a particular dataset. B) Power is plotted as a function of ground truth effect size magnitude, pooling across benchmarking of all datasets. First, the ground truth effect size magnitudes were averaged with a sliding window approach with window size equal to 0.1 (Cohen’s d) and 75% overlap. Then, for each method (indicated by color), the power for the components (either edges or networks) corresponding to the effect size magnitudes within a given window were averaged and plotted on the y-axis. C) Histograms demonstrating the relative occurrence of ground truth effect size magnitudes. Effect size magnitude distributions are again pooled across all datasets. These are shown at the 35 edge-centric network level, the 55 node-centric network level, and the edge level. Note that we set the x-axis to artificially stop at 0.8 because all distributions past that point were near-zero.

Next, we calculated power for each combination of dataset, subsample size, and number of edge-centric networks (for ecNBS) to reveal the relationship between these factors and power. A major factor contributing to statistical power for ecNBS was subsample size, with mean power increasing as subsample size increased for any number of edge-centric networks used (see the first six bars in Fig. 3A). Another prevalent factor contributing to statistical power was the number of edge-centric networks used in the ecNBS algorithm; using more networks with ecNBS tended to reduce the mean power. This trend was also largely revealed in the mean power for benchmarking on each dataset separately, even in the HBN and PNC datasets which had much lower mean power in all cases. The final factor contributing to statistical power investigated here was the dataset for which benchmarking was performed. Mean power was always highest for the PNC age dataset, second highest for the HCP sex dataset, third highest for the HCP IQ dataset, and indistinguishably low for the HBN and PNC handedness datasets. Taken together, these results suggest that ecNBS performs as expected within a dataset, regardless of the number of edge-centric networks used in ecNBS or subsample size. Comparing across datasets, the results indicate that perhaps the distribution of effect sizes throughout the connectome influences the performance of ecNBS. However, edge-level effect sizes did not drive this alone; for example, the HCP IQ edge-level effect sizes (SI Fig. 2) were lower than those of the PNC handedness dataset (SI Fig. 4), yet power was higher in the IQ dataset, most notably when using 35 edge-centric networks and 40 subjects. This suggests that the baseline edge-level effect sizes are uniquely modulated by the edge-centric network partition to differentially affect performance across datasets.

### 3.3. Relative performance of ecNBS varies with effect size and sample size

Even though methods like NBS and edge-level + FDR are able to reveal significance of effects at the edge level, whereas ecNBS and cNBS can only reveal significance of effects at the network level, reporting effects at the edge level may not be the most important characteristic to consider when choosing a method to use for statistical analysis. Therefore, we compared the performance of ecNBS with those of the previous methods. Edge-level + FDR had the lowest power with NBS following; hence, ecNBS outperformed both edge-level methods. Finally, ecNBS always had a higher mean power than did cNBS, demonstrating that among the network-level methods, ecNBS was the best choice for these datasets.

We then pooled the component-level power across all datasets such that we could also pool effect sizes to obtain a better effect size distribution. Using a sliding window, we averaged nearby effect size absolute values and the corresponding power values to demonstrate the relationship between a component’s ground truth effect size magnitude and its detection rate for each analysis method and subsample size (Fig. 3B). The most striking feature of these plots was that no method consistently achieved a mean component-level power above 80% for most effect size magnitudes, even with the largest sample size tested. As 80% is often set as the threshold for sufficient power, this poses a concern for analyzing functional connectivity datasets in this way when the effect sizes are less than 0.9019, the largest effect size seen for these phenotypes in the current datasets.

Comparatively, any ecNBS method was the most powerful for most effect size magnitudes and subsample sizes tested here, with cNBS and NBS rarely outperforming ecNBS. Conversely, edge-level + FDR was the lowest-performing correction method for all effect sizes and the lowest two sample sizes measured here, only outperforming NBS slightly in the middle range of effect sizes for the largest subsample size. If, for example, ecNBS with 35 edge-centric networks is not twice as powerful than cNBS for each effect size magnitude, how can ecNBS be more than twice as powerful on average? And how can NBS and edge-level + FDR have such low average power even though they can attain substantial power at higher effect size magnitudes? Questions like these can be answered by the component-level effect size magnitude distributions (Fig. 3C), which are shown pooled across datasets. Because ecNBS with 35 edge-centric networks was the best-performing among the ecNBS variants, we only plotted the histogram for those edge-centric networks. For a full visualization of effect size distribution for all network partitions as well as the connectome-wide distribution for each dataset, refer to SI Figs. 1-5. Compared to the node-centric network-level effect sizes, the 35 edge-centric network-level effect sizes were skewed towards larger magnitudes, allowing ecNBS with 35 edge-centric networks to attain a much higher power on average. Conversely, the majority of the edge-level effects were near-zero, explaining the resulting average power for NBS and edge-level + FDR. Thus, these examples explain the discrepancy between relative performance with regards to effect size magnitude and absolute performance for an entire distribution of effect sizes. Furthermore, these results highlight the importance of understanding the regime in which the phenotype of interest resides in terms of effect size distribution and how this impacts the sample size one should choose. For existing datasets, it helps inform the researcher which correction method or combination of methods one should select for statistical analysis.

### 3.4. ecNBS is robust to choice of clustering algorithm and atlas

To determine whether the results for power and false positive detections change with different choices of clustering algorithm, we performed benchmarking of ecNBS on the HCP sex dataset using k-means rather than Normalized Cut to determine the edge-centric networks. Although mean power was much lower for each network partition in this case, power was still higher than that of cNBS, NBS, and edge-level + FDR (SI Fig. 6A). Additionally, the same trends in power were observed for this case as were observed for networks constructed with Normalized Cut. Similarly, ecNBS with k-means networks controlled all three metrics of FPR comparably to ecNBS with Normalized Cut networks (SI Fig. 6B-D).

To determine whether the results for power and false positive detections change based on the atlas used to process the connectomes, we performed benchmarking for all inferential methods on the HCP sex dataset processed with the Shen368 atlas rather than the Shen268 atlas. Mean power was slightly lower for each network-level method and was approximately the same for the edge-level + FDR and NBS (SI Fig. 7A). Relatively, the same trends were followed as compared to benchmarking performed on the data processed with the Shen268 atlas. Finally, all three metrics of FPR were controlled comparably to that of the main analyses (SI Fig. 7B-D).

### 3.5. Edge-centric and node-centric networks capture different aspects of underlying edge-level effects

The main purpose of using knowledge of the large-scale network architecture of the brain to control false positive detections is to summarize the connectome by networks with the assumption that edges within a predefined network share similar effect sizes. Necessarily, a null hypothesis can never be rejected at the edge-level using network-level methods such as cNBS and ecNBS. Yet, it can still be informative to extrapolate to the edge level, especially in circumstances in which the predefined edge-centric networks do not align with node-centric networks. Thus, the ideal scenario for using cNBS or ecNBS would be one in which all edges within a network have the same ground truth effect size, leading to the most accurate results with respect to the single edge level. Because cNBS uses networks defined at the node level to group edges, it can potentially ignore crucial edge relationships. Therefore, we leveraged correlations between edges across subjects in an independent dataset to assign edges to edge-centric networks for use in ecNBS. The question remains as to how well each method for network construction captures the underlying edge information.

**Fig. 4.**
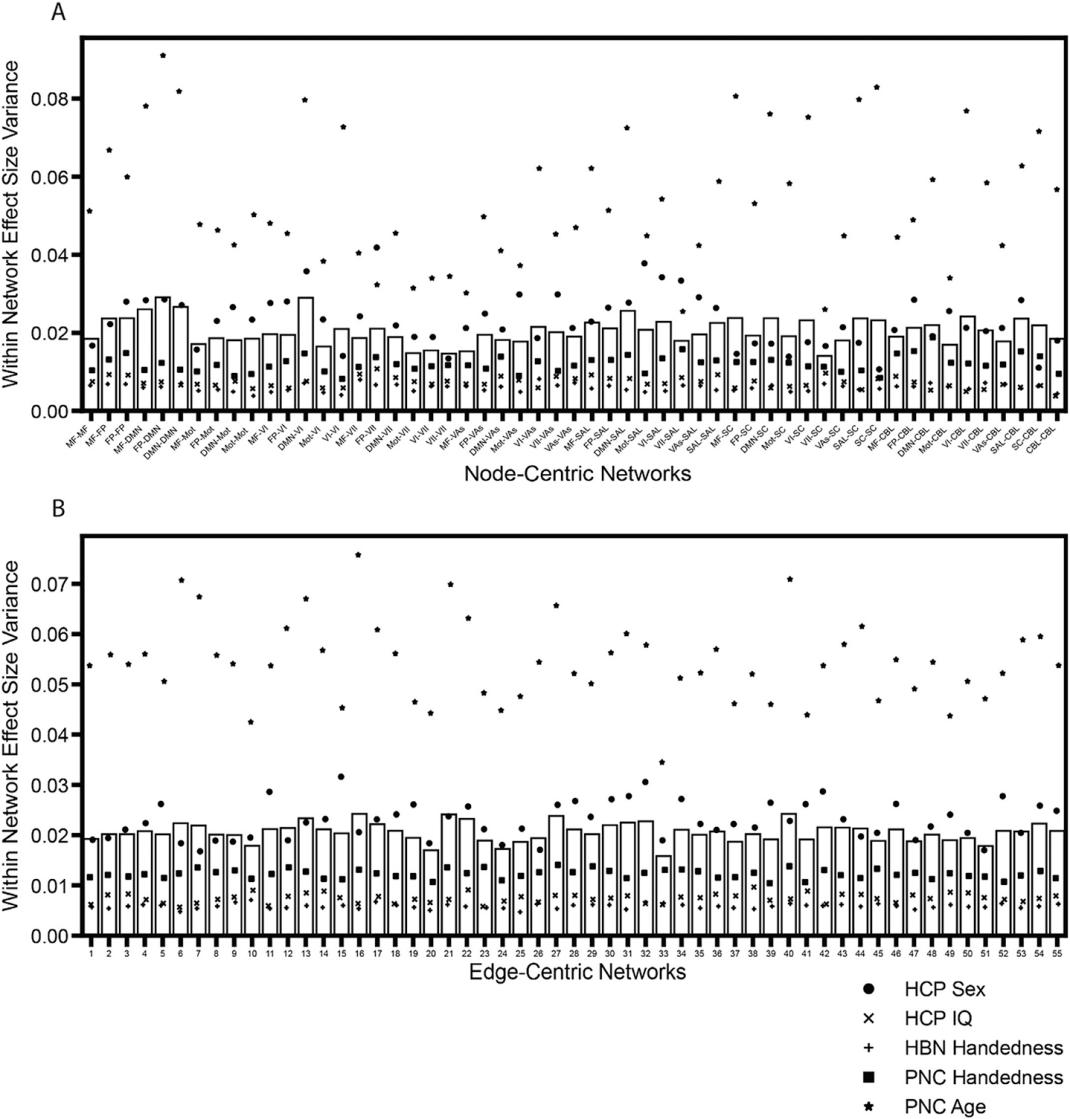
Mean within-network effect size variance across datasets. For each network, variance was taken across the constituent edge-level effect sizes for each dataset and averaged across datasets. A) Node-centric networks used in cNBS with the corresponding network labels. B) 55 edge-centric networks used in ecNBS with network number labeled (label is arbitrary).

To address this question, we calculated the within-network edge-level effect size variance for node-centric networks and edge-centric networks (Fig. 4 and SI Figs. 8 and 9). This variance measures how similar within-network edges are to each other, serving as a metric of how suitable it is to simply combine within-network edges for statistical analysis; if a network has a high effect size variance, it is less homogeneous and therefore less suitable for combining edges within-network. Here, we demonstrated that the within-network homogeneity of edge-level effect sizes across datasets was more variable across networks when using node-centric networks than when using the same number of edge-centric networks (Fig. 4). Notably, these edge-centric networks had a similar size range to that of the node-centric networks, implying that network size distribution does not underlie the effects observed here (SI Fig. 10). In the node-centric partition, the within-network variance was particularly high in the network that contains edges between the frontoparietal and DMN networks and in the network that contains edges between the DMN and VI networks. On the other hand, the within-network variance was lower in the network that contains edges between the VII and subcortical networks and in the network that contains edges that reside completely within VII. The result is that any network-level detection with cNBS of the lower-homogeneity networks is not as true to the underlying edge-level effects as compared to a network-level detection of a higher-homogeneity network. Using edge-centric networks seems to alleviate this potential bias, with apparently lower variability of within-network effect size homogeneity across networks. However, in individual datasets the node-centric networks were found to have similar, if not slightly lower, median within-network effect size variance (SI Figs. 11 and 12), indicating that node-centric networks can be astonishingly good, and even better on average, at representing their constituent edges despite not being constructed with edge information. Therefore, depending on the dataset, edge-centric networks may be more fair across networks even though node-centric networks may be better on average, indicating that there is a trade-off between the two methods for assigning edges to networks.

### 3.6. Detections in a large dataset

**Fig. 5.**
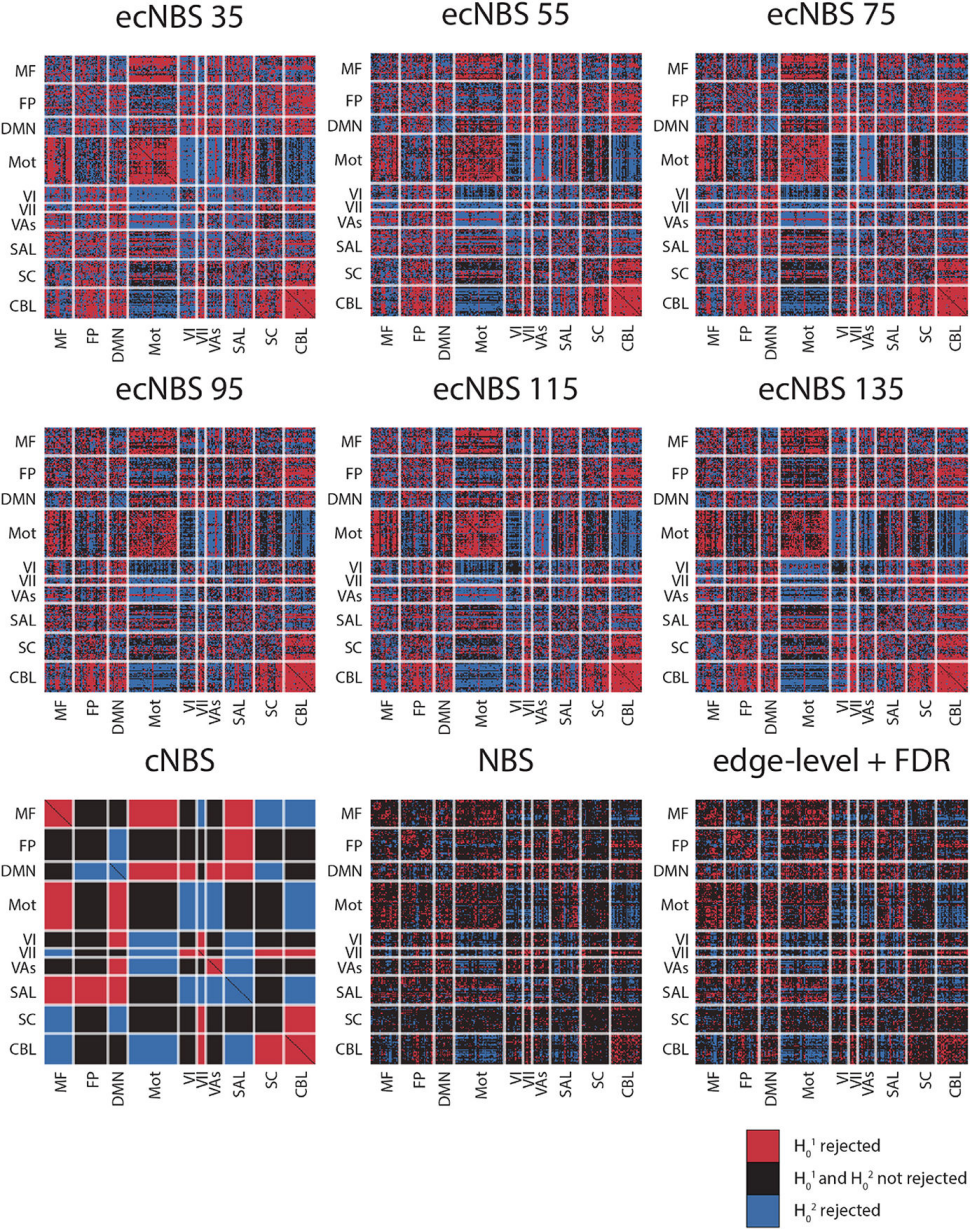
Detections on the full sample of HCP sex data with a two-tailed t-test. Each matrix reflects the results performed with different inferential methods with the labels reflecting which method was used. Shown here are component-wise rejections of the null hypotheses. Red indicates that the null hypothesis that the component is not greater in males than in females (H_0_^1^) was rejected with *α* = 0.025. Blue indicates that the null hypothesis that the component is not greater in females than in males (H_0_^2^) was rejected with *α* = 0.025. Black indicates that neither null hypothesis was rejected.

Finally, we wanted to compare the methods in terms of what results they produce for a full dataset. To this end, we implemented each of the methods for the full HCP sex dataset (*n*=1058) and performed a two-tailed t-test, noting all component-level rejections of the null hypotheses, denoted as detections (Fig. 5). Just as power was higher for ecNBS compared to the other methods, so too were detections, with the number of detections decreasing with increasing number of edge-centric networks used. Additionally, ecNBS appeared to detect edge-centric networks that included edges widely distributed across node-centric networks. Of course, although inferences cannot be made at the edge-level when using network-level methods, it is interesting to note the distribution of detected edges throughout the connectome. Again following the previously shown trend, cNBS procured the next-highest number of detections. However, differing from this trend, it was demonstrated that edge-level + FDR actually procured more detections than did NBS. For both NBS and edge-level + FDR, detections appeared particularly low in subcortex-or-cerebellum-related node-centric networks. Overall, these results highlight the overall power that is delivered by network-level methods while simultaneously highlighting the ability for methods like edge-level + FDR and NBS to offer more specific, fine-grained details at the edge-level at the expense of detection power.

## 4. Discussion

In the present work, we introduced ecNBS, a method to correct for multiple comparisons that combines networks based on edge information (Gu et al., 2017), with cNBS (Noble, 2022; Noble and Scheinost, 2020). We used a benchmarking process that runs statistics on subsamples of larger datasets and assumes effect sizes from the full dataset represent the ground truth to evaluate performance of our new method, ecNBS, and compared it to existing methods like edge-level + FDR, NBS, and cNBS. ecNBS satisfactorily controlled the network-level FPR for five datasets at three different sample sizes. Motivated by the possibility for edge-centric networks to more fairly cluster edges into networks than node-centric networks, we found that ecNBS offers substantial improvements in power over other methods. For example, with a sample size of 120 subjects and using 35 networks, ecNBS was ~16 times more powerful than NBS and ~3 times more powerful than cNBS in the HCP sex dataset. Notably, this result was due to a combination of increased power at each effect size and higher network-level effect size distributions.

The principal novelty of this work lies in the use of edge-centric networks to make network-level inferences of various phenotypes. That edge-centric networks differ for many phenotypes and across many datasets corroborates previous conclusions of the utility of edge-centric information and adds to the growing list of applications for edge-centric data (Chumin et al., 2022; Esfahlani et al., 2021; Faskowitz et al., 2022, 2021, 2020; Jo et al., 2021a, 2021b; Sporns et al., 2021). This is important because in the context of functional connectomes, edges are only an abstract notion of connectivity between brain regions and do not require a simple physical or biological basis. Thus, although we do not yet have a complete understanding or a comprehensive interpretation of edge-centric networks, their repeated correspondence with phenotypic measures indicates their broader applicability and suggests that an exciting field of edge-centric research remains ripe for future exploration, hopefully leading to the discovery of a consistent interpretation of edge-centric connectivity.

In addition to comparing various statistical correction methods in terms of detection power, we also compared the variability of edge-level effects in edge-centric networks to node-centric networks. In contrast to our hypothesis, we demonstrated that although the edge-centric networks are more evenly representative of their constituent edges, node-centric networks have a comparable or even lower average within-network effect size variability. This was surprising because the node-centric networks were able to achieve this without explicitly incorporating edge-level similarity during their construction (Horien et al., 2020), reflecting a level of correspondence between edge- and node-level information as was previously identified in a different edge-centric method (Faskowitz et al., 2020; Jo et al., 2021b; Novelli and Razi, 2022). This effect was so apparent that for most individual datasets, the median within-network variance across edge-centric networks was higher than that of the node-centric networks. However, the edge-centric networks excelled in maintaining a similar level of within-network effect size variance across networks, whereas the within-network variation for node-centric networks was often unevenly distributed between the networks.

The simplest explanation is that edge-centric networks are not “better” than node-centric networks. Alternatively, we propose a more nuanced version of this explanation; the presently used edge-centric networks may not be better, but with more time, we predict that edge-centric networks may fully overtake node-centric networks. The node-centric networks we used in this work were first implemented in 2015 (Finn et al., 2015), almost one decade ago. Furthermore, many functional networks like the default mode, frontoparietal, and salience have been widely studied with functional connectivity for nearly 20 years. Hence, the concept of functional networks has already become deeply ingrained in neuroimaging research. By contrast, an edge-centric perspective of the functional connectome (Faskowitz et al., 2020) only dates back a couple of years. As such, there has been less time to optimize the process for uncovering edge-centric networks and determine the optimal parameters for which to create an appropriate number of edge-centric networks. There has also been less time to begin to interpret the meaning of edge-centric networks; this coupled with the fact that edges, unlike nodes, have no clear physical basis in the brain complicates the endeavor to refine the process for creating and basis for interpreting edge-centric networks. Additionally, edge-centric networks were found to be largely unaligned to node-centric networks (SI Fig. 13), further emphasizing the distinction between the two types of networks. Therefore, given that ecNBS was already demonstrated to perform well, we hypothesize that as these processes and interpretations are refined, edge-centric networks will better encapsulate the underlying edge-level effects and ecNBS performance will improve greatly. Overall, we envision a promising future for ecNBS in the realm of statistical inference at the network level. Yet, there are still many factors to consider—especially given the early stages of edge-centric research—when deciding which method to use for inference, as each type of network may excel in different scenarios.

It should also be noted that with the appropriate datasets and phenotypes, ecNBS may not be the most powerful method. As a case example, recall that the mean power was higher for the IQ dataset than for the PNC handedness dataset when using the network-level methods even though the edge-level effect sizes were larger in the handedness dataset. This example illustrates that the network partition itself affects the resulting power, semi-independent of the underlying edge-level effects, such that the effect size distribution at the network level is intrinsically linked to the topological distribution of edge-level effect sizes. This is reasonable when considering the fact that networks were constructed by either edge-edge or node-node similarity information rather than effect size similarity. So although the assumption is that the edge-centric networks would more accurately portray the underlying edge-level effects than would the node-centric networks, we revealed that even this distinction is entirely dependent on the dataset being used. Other studies have noted that the network description of the functional connectome is inconsistent across studies (Uddin et al., 2019), that brain state and individuality influence network organization (Salehi et al., 2018), and that networks may even reorganize within a single scan (Iraji et al., 2019), and with a range of effects that can be tested, it is only logical to assume that some effects would not be well categorized by the presently-used edge-centric networks. In such a case, it may lead to relatively higher power to use cNBS or even NBS or edge-level + FDR instead of ecNBS.

Complicating the matter is the factor of sample size; since we only performed benchmarking for sample sizes of 40, 80, and 120 subjects, it is unknown whether with a larger sample size there would be some crossing point at which the other methods become more powerful than ecNBS for a given dataset. For example, with increasing sample size, the plots of effect size magnitude versus power for edge-level + FDR increased at a faster rate than any other method, indicating that perhaps there exists a sufficiently large sample size such that we would observe such a crossing point. Additionally, when using the full sex dataset, edge-level + FDR had more detections than did NBS, in contrast to the mean power results, further supporting the notion that sample size may differentially impact the different methods. However, in neuroimaging, typical sample sizes tend to be even lower than 40 subjects (Poldrack et al., 2017; Szucs and Ioannidis, 2020), meaning that even if this crossing point exists for these datasets or others, it is irrelevant if the field does not shift to experiments with larger samples. Therefore, primarily due to differing topological distributions of effect sizes across datasets and the factor of sample size, it becomes complicated to prescribe with certainty which method will be most-suitable for any given study; the most prudent option is to then use various methods depending on whether the hypothesis is at the edge- or network-level and to compare across to gain a more complete understanding of the dataset at hand.

Lastly, we address the potential limitations of our methodology. One limitation was that our results are directly related to the phenotypes and datasets that were studied; hence, our results cannot be indicative of the results one could obtain with any other dataset or phenotype. However, as we did study five phenotypes across three datasets, we believe the results stem from a diverse-enough study such that some generalizability can be assumed. A second limitation was that although the edge-centric networks worked well for inferencing, they did not outperform the node-centric networks at the level initially hypothesized due to their edge-specific information. This provides an opportunity for future work to interpret the meaning of edge-centric networks and how the underlying information relates to that of node-level information. Relatedly, a third limitation was that edge-centric networks are relatively new and have not yet been optimized in their construction. Looking forward, this represents a strength because as edge-centric networks are improved and become more understood, the method presented here will become easier to implement and detected group differences will be more interpretable. Similarly, a fourth limitation is that we only implemented one method to determine pairwise edge similarity, even though other methods exist. Although the use of other methods would change the interpretation of the resulting edge-centric networks, they would be equally interesting to implement in future studies. A fifth limitation is that it has recently been demonstrated that edge functional connectivity can often be faithfully recovered from node functional connectivity (Novelli and Razi, 2022). But, we regard this as impacting the interpretation of the meaning of edge-centric networks rather than invalidating the use of such networks to improve statistical inference. A sixth potential limitation was that we only used one atlas for all of the main analyses to convert voxel time series to lower-dimensional node time series. However, based on our replication of the main results using data processed with the Shen368 atlas, we believe that the results are likely to hold for other atlases. A seventh limitation was that our node-centric and edge-centric networks were derived from an independent dataset rather than information from the data. At the present, it is unknown whether so-called canonical mappings are truly generalizable across datasets, but we argue that using the independent dataset avoids any potential statistical bias that could be introduced if constructing edge-centric networks with the same dataset being compared for statistical differences. The existence of this bias would be an interesting topic of study to confirm whether it is best to use an independent dataset for edge-centric network construction. Finally, we only implemented a Normalized Cut algorithm to construct edge-centric networks for all of our main analyses, which could influence the results. However, in practice any network-constructing method could be employed, and we contend that the results would be similar based on our benchmarking results using k-means networks.

### 4.1. Conclusion

In conclusion, the study at hand introduces a promising new statistical method for analyzing human functional connectomes, ecNBS. Within the constrained network-based statistic category, ecNBS offers substantial improvements over the original cNBS but shares the same restriction in being unable to make inferences at the edge level. We therefore view this new method as a way to explore edge-centric hypotheses at the network level as studies continue to explore edge-edge relationships.

## Supplementary Material

1

## Data Availability

I have shared the links to my data and code in section 2.8 of the Methods in the article.
