# Supplementary material for: Leveraging edge-centric networks complements existing network-level inference for functional connectomes
Source: Neuroimage. Author manuscript; Available in PMC 2023 Jan 13. (PMC9838718; doi:10.1016/j.neuroimage.2022.119742)
Supplement: 1 [file NIHMS1860181-supplement-1.docx]

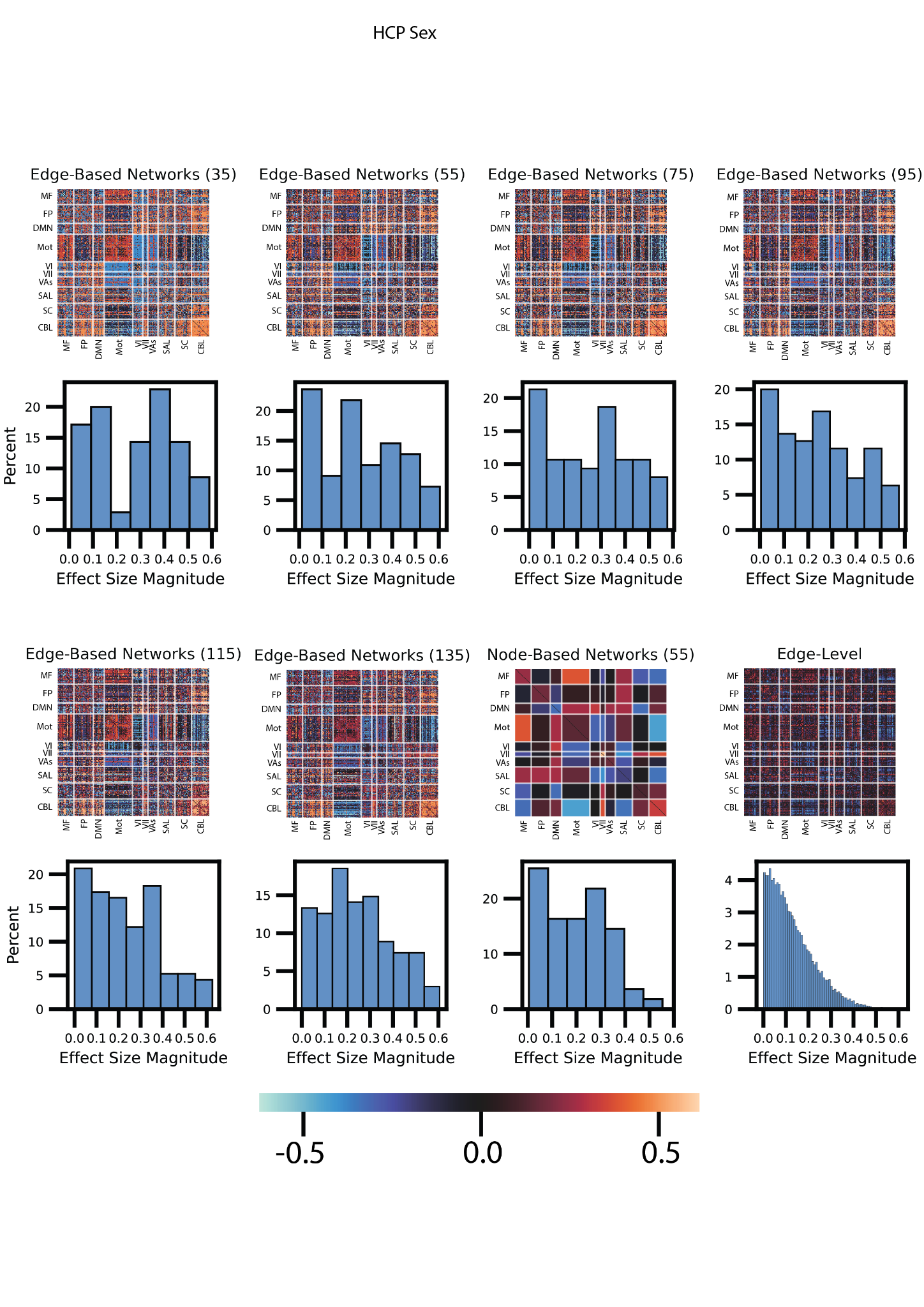


**Figure S1:** HCP sex dataset effect sizes. For each network parcellation as well as for the edge level, connectomes of effect size were reorganized and plotted by subnetwork. Additionally, the effect size magnitude histogram was plotted for each network parcellation as well as for the edge-level effect sizes. The colorbar corresponds to values across all connectomes.


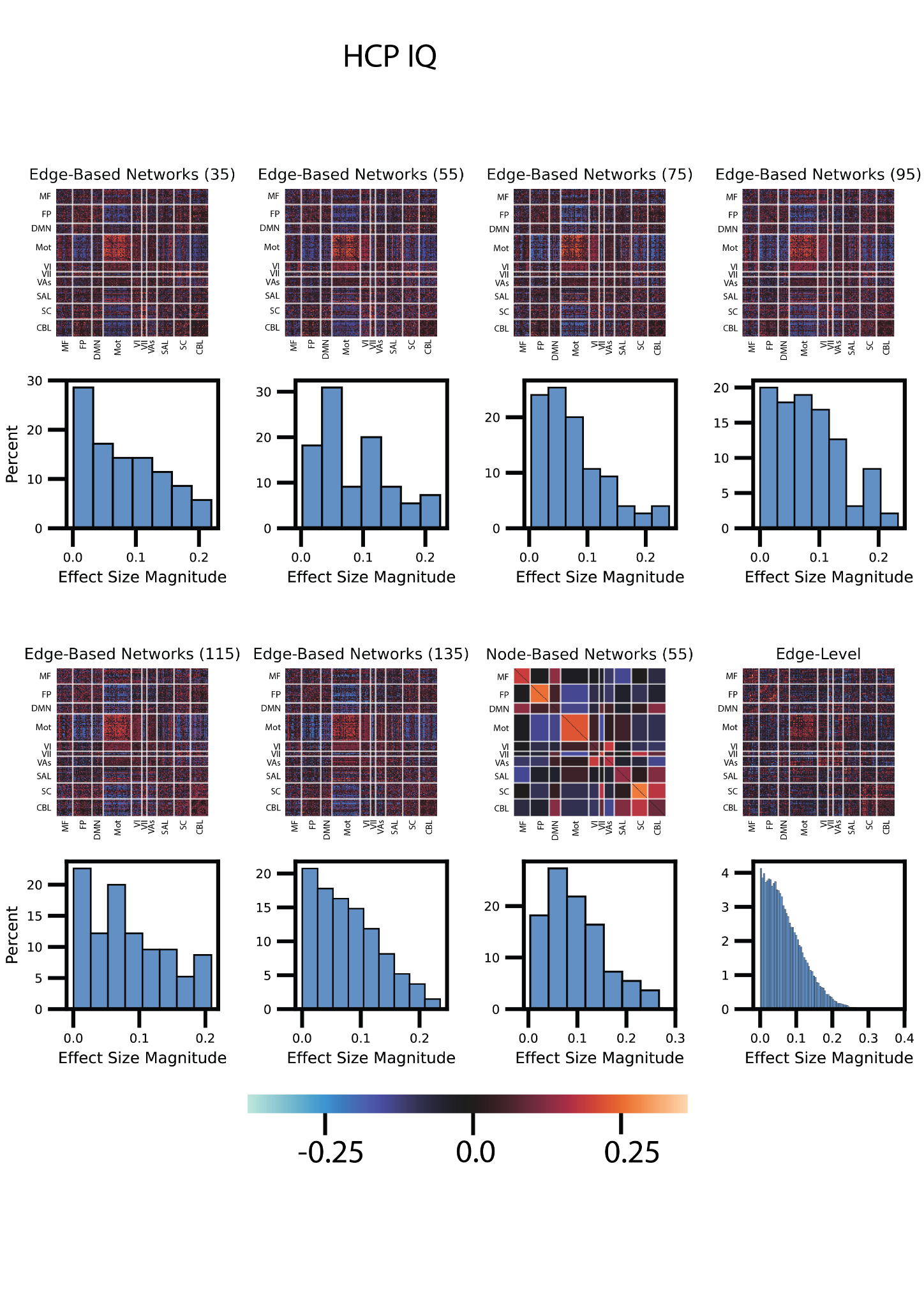


**Figure S2:** HCP IQ dataset effect sizes. For each network parcellation as well as for the edge level, connectomes of effect size were reorganized and plotted by subnetwork. Additionally, the effect size magnitude histogram was plotted for each network parcellation as well as for the edge-level effect sizes. The colorbar corresponds to values across all connectomes.


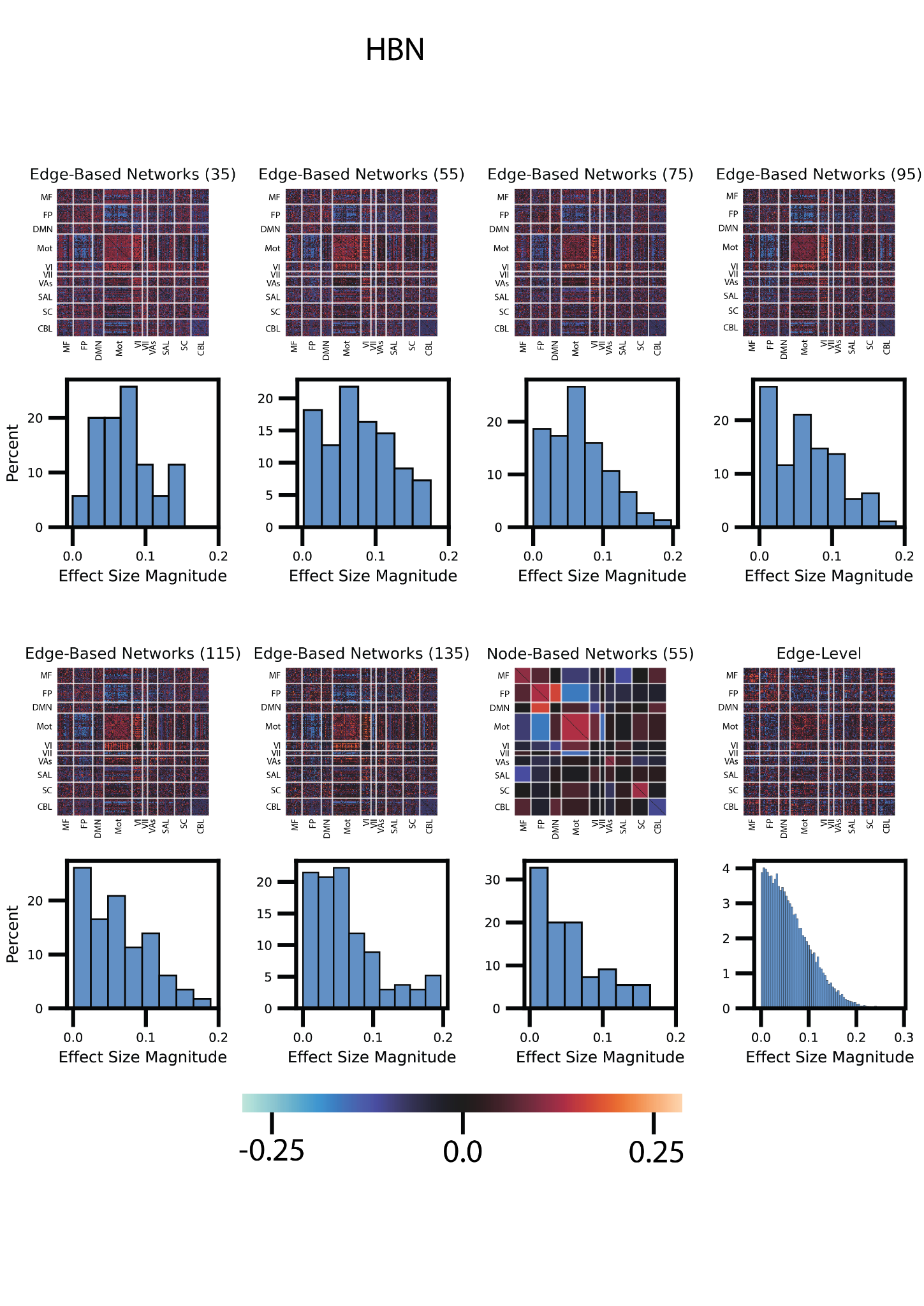


**Figure S3:** HBN handedness dataset effect sizes. For each network parcellation as well as for the edge level, connectomes of effect size were reorganized and plotted by subnetwork. Additionally, the effect size magnitude histogram was plotted for each network parcellation as well as for the edge-level effect sizes. The colorbar corresponds to values across all connectomes.


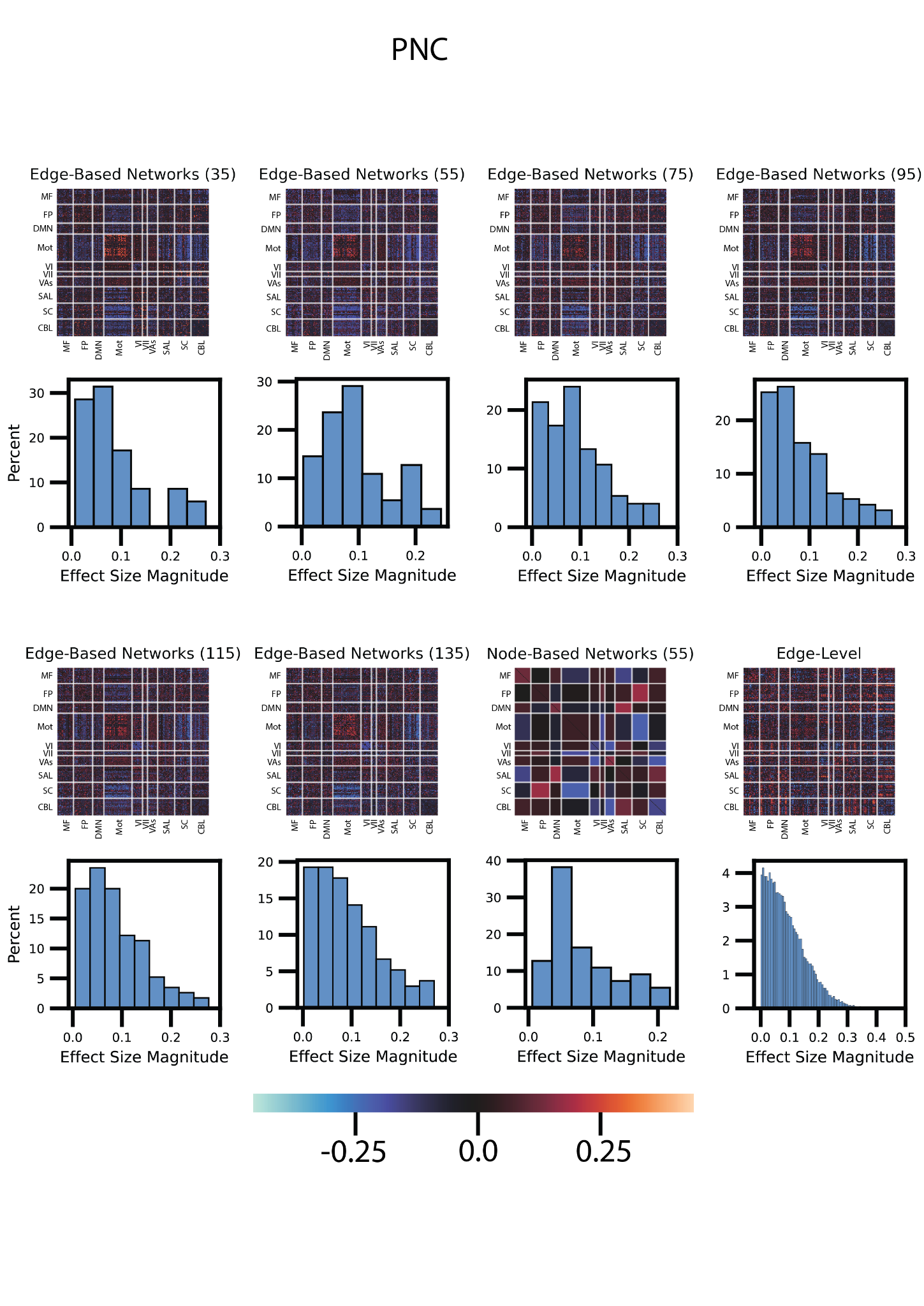


**Figure S4:** PNC handedness dataset effect sizes. For each network parcellation as well as for the edge level, connectomes of effect size were reorganized and plotted by subnetwork. Additionally, the effect size magnitude histogram was plotted for each network parcellation as well as for the edge-level effect sizes. The colorbar corresponds to values across all connectomes.


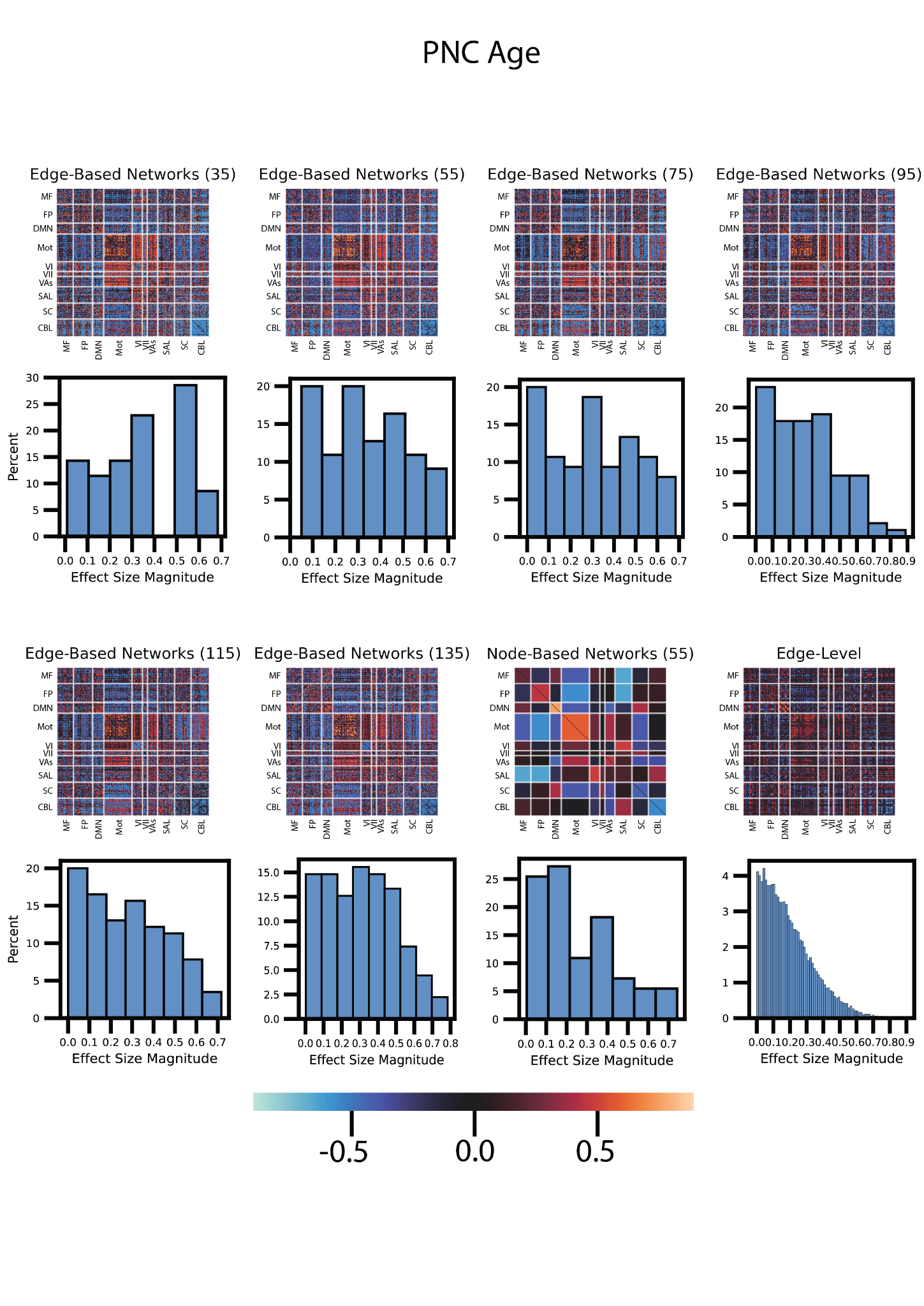


**Figure S5:** PNC age dataset effect sizes. For each network parcellation as well as for the edge level, connectomes of effect size were reorganized and plotted by subnetwork. Additionally, the effect size magnitude histogram was plotted for each network parcellation as well as for the edge-level effect sizes. The colorbar corresponds to values across all connectomes.


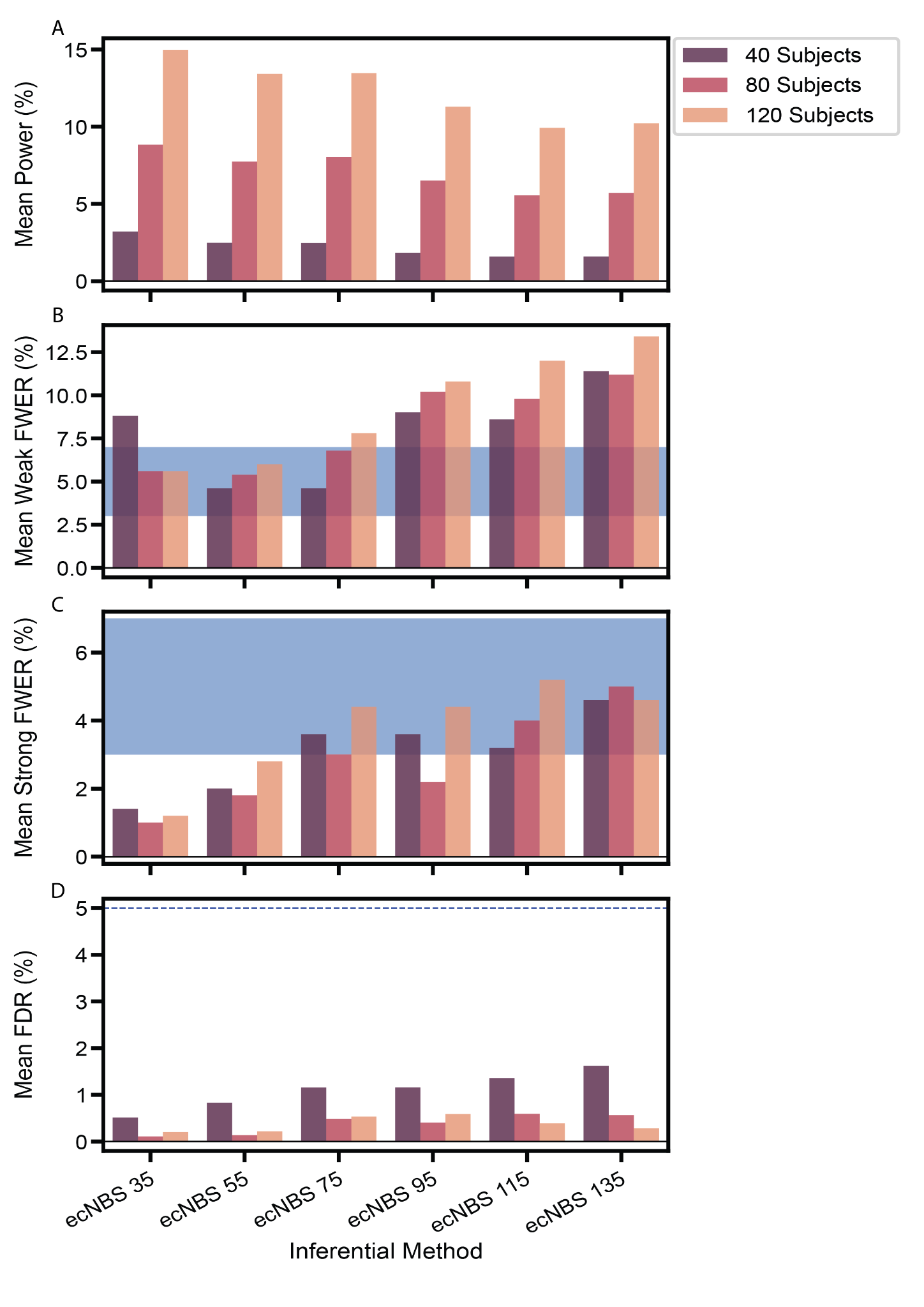


**Figure S6:** Effect of k-means networks on ecNBS performance. **A)** Power is averaged across networks in the HCP sex dataset. Positions of the bars on the x-axis correspond to the method used with benchmarking. Colors correspond to the subsample size. To assess the ability to control the false positive rate, we used **B)** weak FWER, **C)** strong FWER, and **D)** FDR. Each metric was computed at the repetition level and then averaged across benchmarking repetitions. Each set of bars on the x-axis represents an inferential method that was used for benchmarking. The colors of the bars correspond to the sample size used during benchmarking. The shaded blue regions in **B)** and **C)** are the 95% confidence interval around a FWER of 5% for 500 benchmarking repetitions, and the blue line in **D)** is set at 5% to show the permissible FDR.


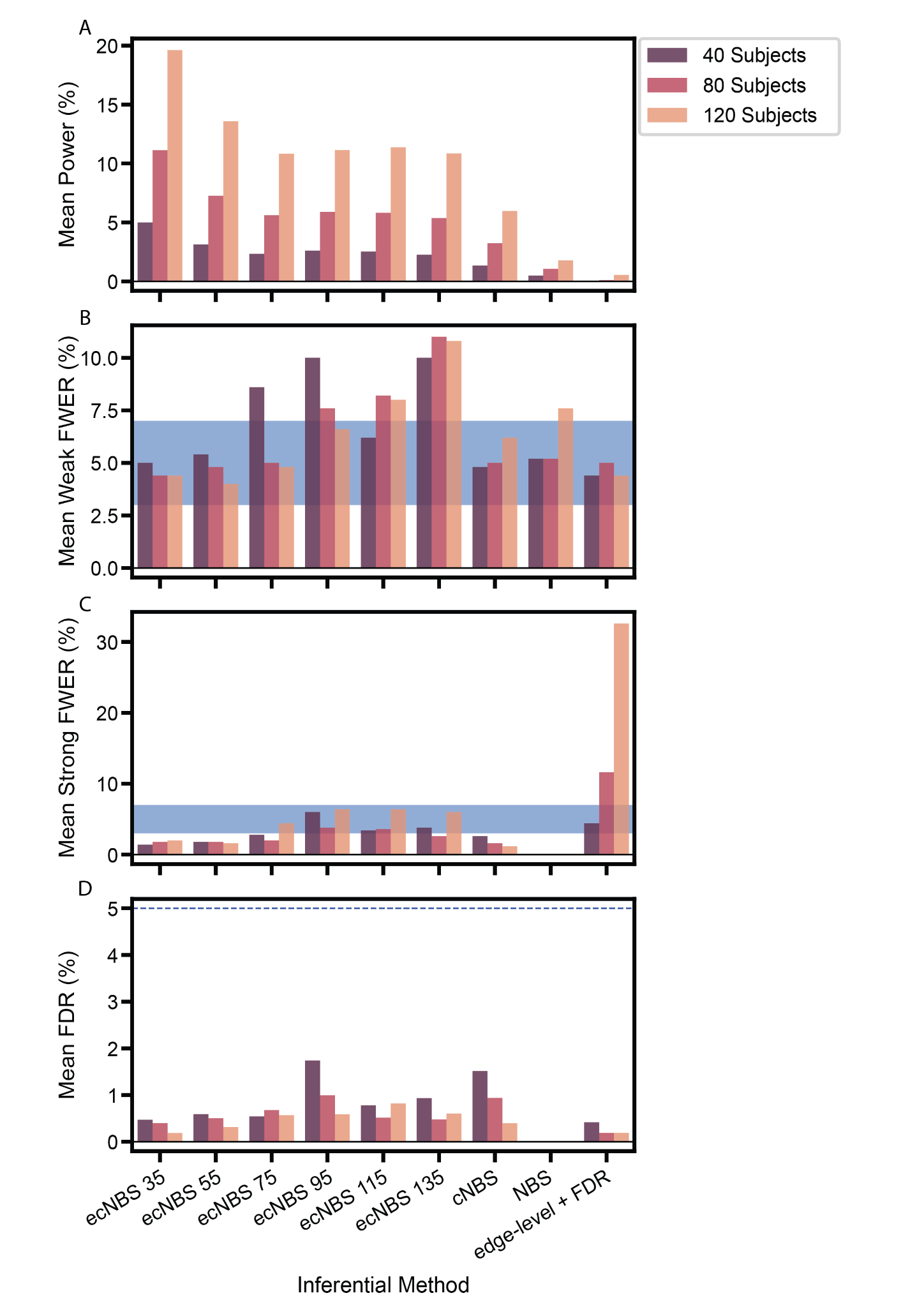


**Figure S7:** Effect of Shen368 atlas on benchmarking performance. **A)** Power is averaged across networks in the HCP sex dataset. Positions of the bars on the x-axis correspond to the method used with benchmarking. Colors correspond to the subsample size. To assess the ability to control the false positive rate, we used **B)** weak FWER, **C)** strong FWER, and **D)** FDR. Each metric was computed at the repetition level and then averaged across benchmarking repetitions. Each set of bars on the x-axis represents an inferential method that was used for benchmarking. The colors of the bars correspond to the sample size used during benchmarking. The shaded blue regions in **B)** and **C)** are the 95% confidence interval around a FWER of 5% for 500 benchmarking repetitions, and the blue line in **D)** is set at 5% to show the permissible FDR.


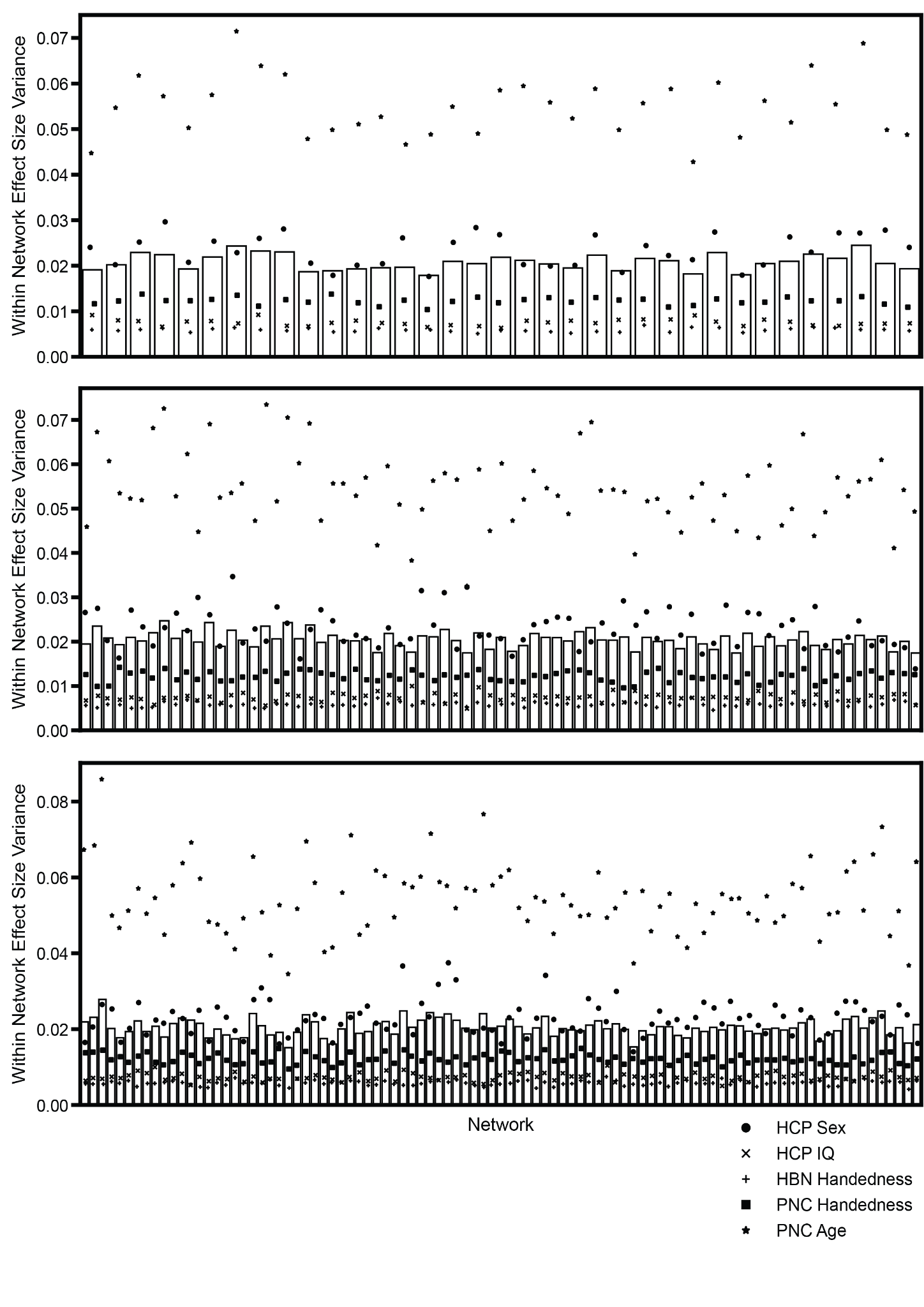


**Figure S8:** Mean within-network effect size variance across datasets. For each edge network, variance was taken across the constituent edge-level effect sizes for each dataset and averaged across datasets. Here, this was performed for each edge-centric network parcellation with 35, 75, and 95 networks.


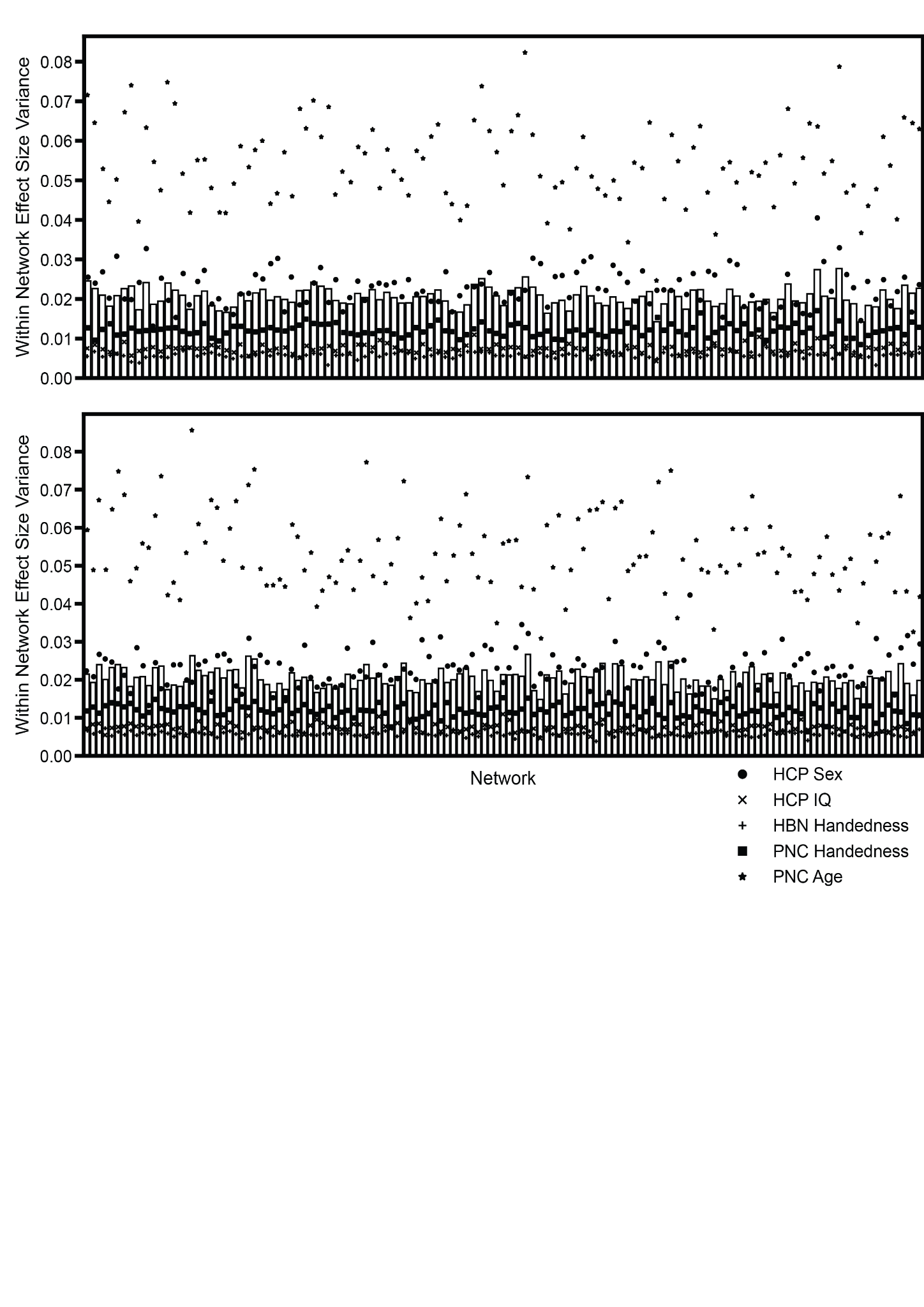


**Figure S9:** Mean within-network effect size variance across datasets. For each edge network, variance was taken across the constituent edge-level effect sizes for each dataset and averaged across datasets. Here, this was performed for each edge-centric network parcellation with 115 and 135 networks.


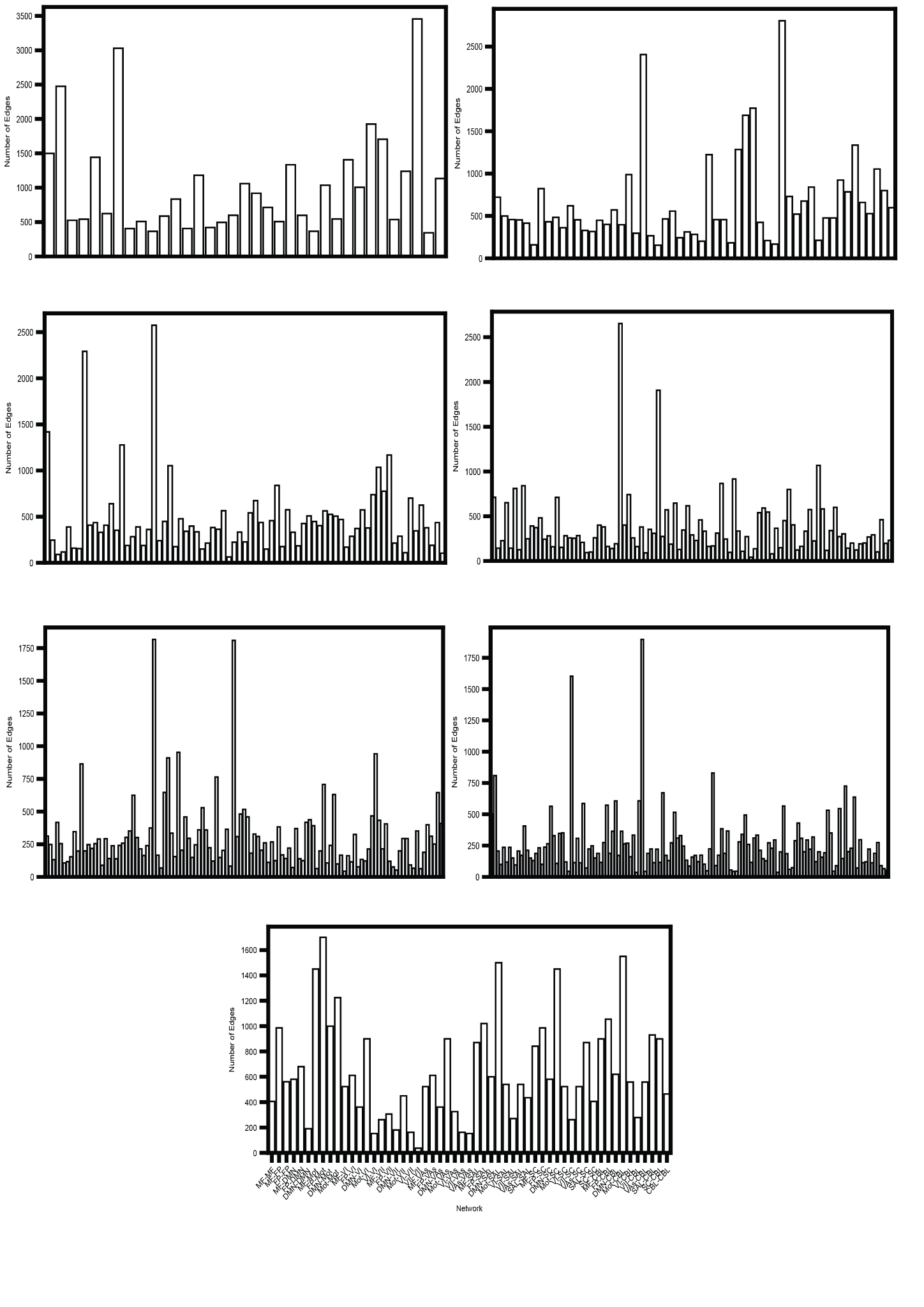


**Figure S10:** Network sizes for each network assignment scheme. Each plot depicts the number of edges in each network for all network assignment schemes. The edge-centric network assignments (first three rows) are unlabeled because the labels are arbitrary. The node-centric network assignments (bottom) are labeled with the subnetwork pairings. Each bar is the number of edges in a given network.


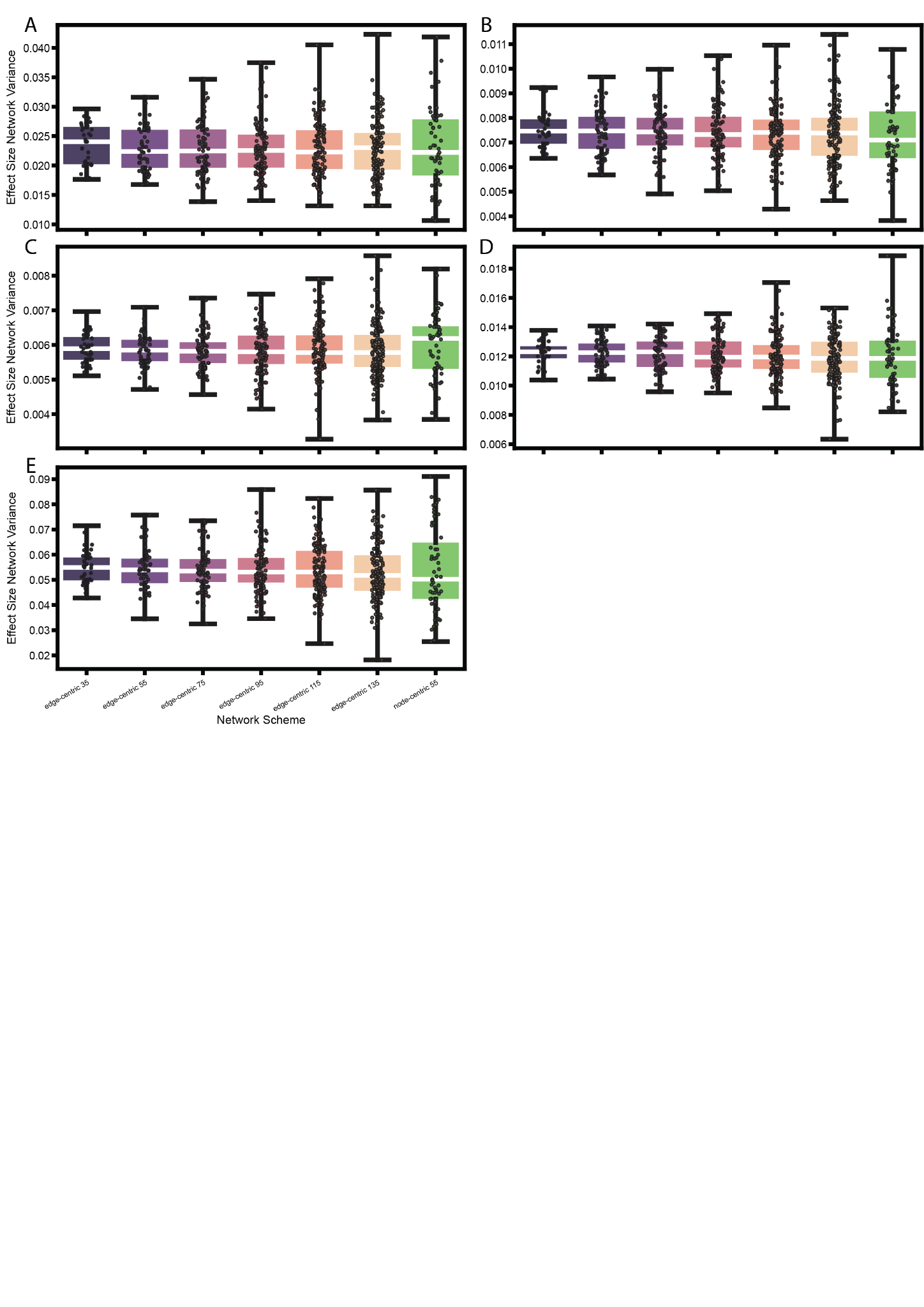


**Figure S11:** Ground truth effect size variance distribution for each network assignment. Each row of this figure reflects benchmarking performed with a different dataset: **A)** HCP sex, **B)** HCP IQ, **C)** HBN handedness, **D)** PNC handedness, and **E)** PNC age. Each boxplot captures the distribution of the variance of ground truth effect sizes within each network in the given network assignment. The points represent the variance of a particular network. The x-position indicates to which network assignment the variances correspond.


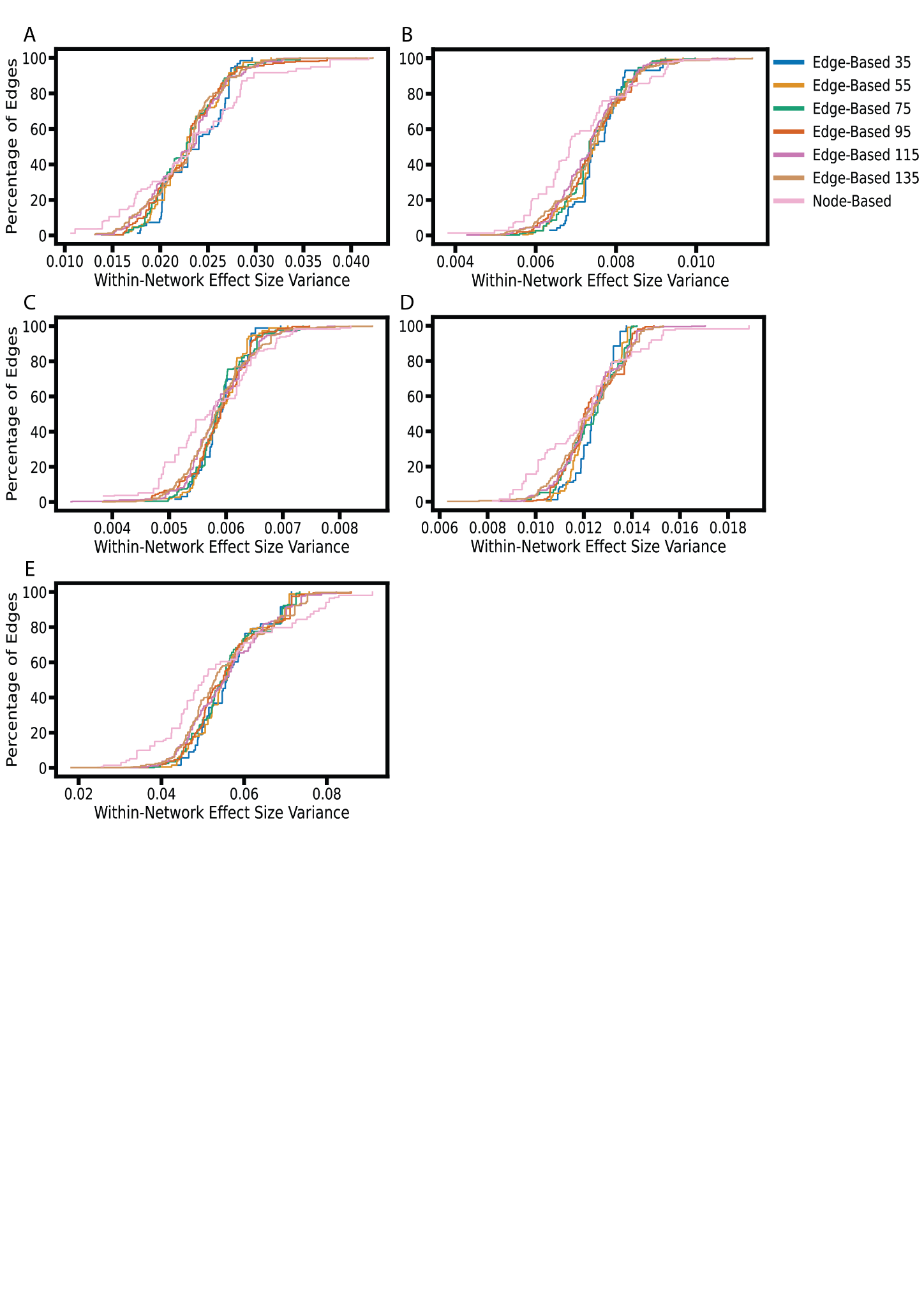


**Figure S12:** Cumulative distribution of within-network effect size variance for each dataset: **A)** HCP sex, **B)** HCP IQ, **C)** HBN handedness, **D)** PNC handedness, and **E)** PNC age. Each point on the x-axis is the within-network variance for a network based on the increasing sort order. The y-axis then describes the percentage of edges that reside in a network with a within-network variance no greater than the corresponding point on the x-axis.


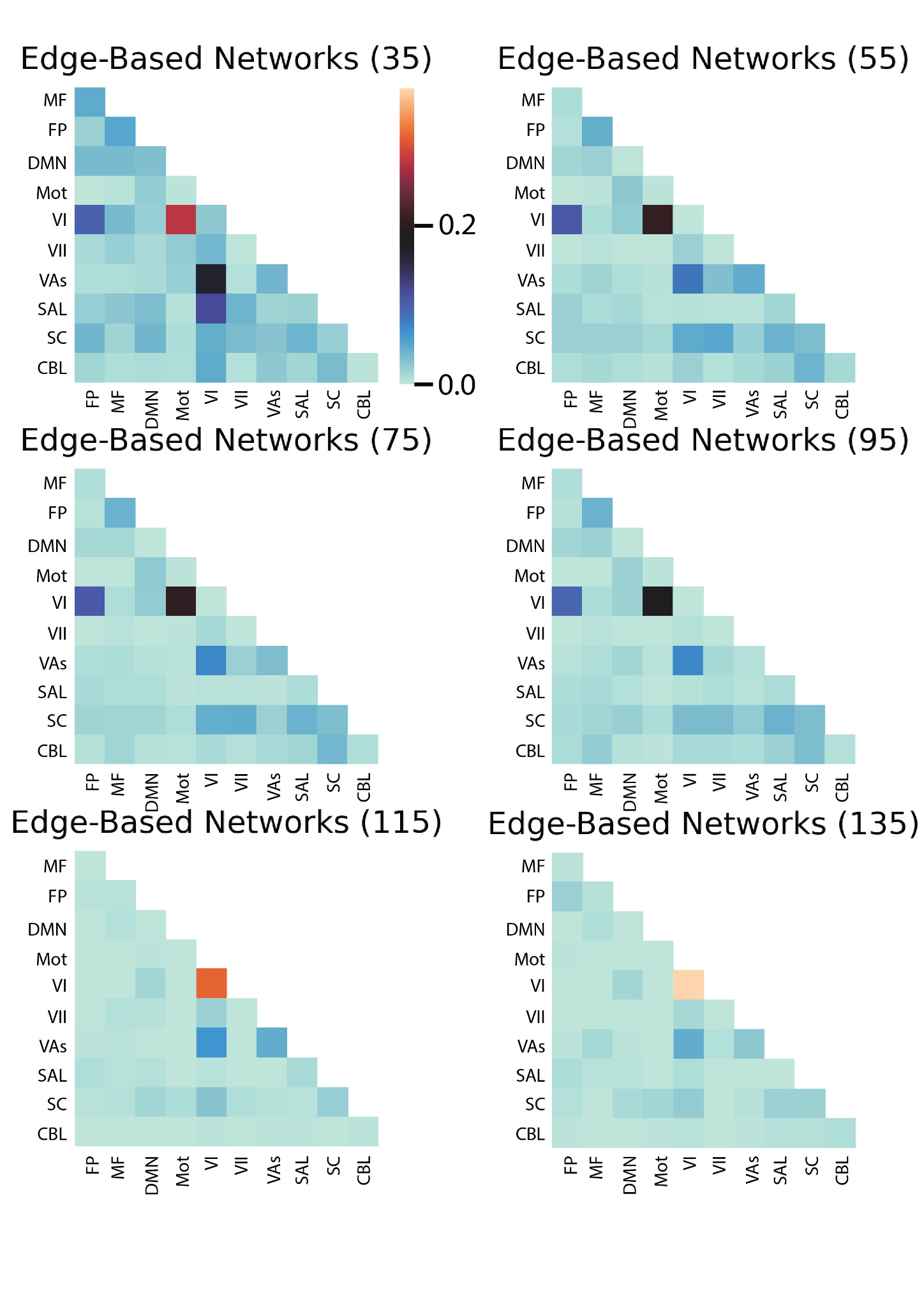


**Figure S13:** Top network overlap between each edge-centric network scheme and the node-centric network scheme. For each edge-centric network scheme, the Jaccard similarity was computed between pairs of edge-centric and node-centric networks. The edge-centric network that was most similar to a node-centric network was taken. Then, for all node-centric networks, the fraction of its edges that are also in that edge-centric network was calculated. Thus, in any of these plots, a matrix value is the fraction of edges in a particular node-centric network that are also present in the edge-centric network that is most similar to any node-centric network.


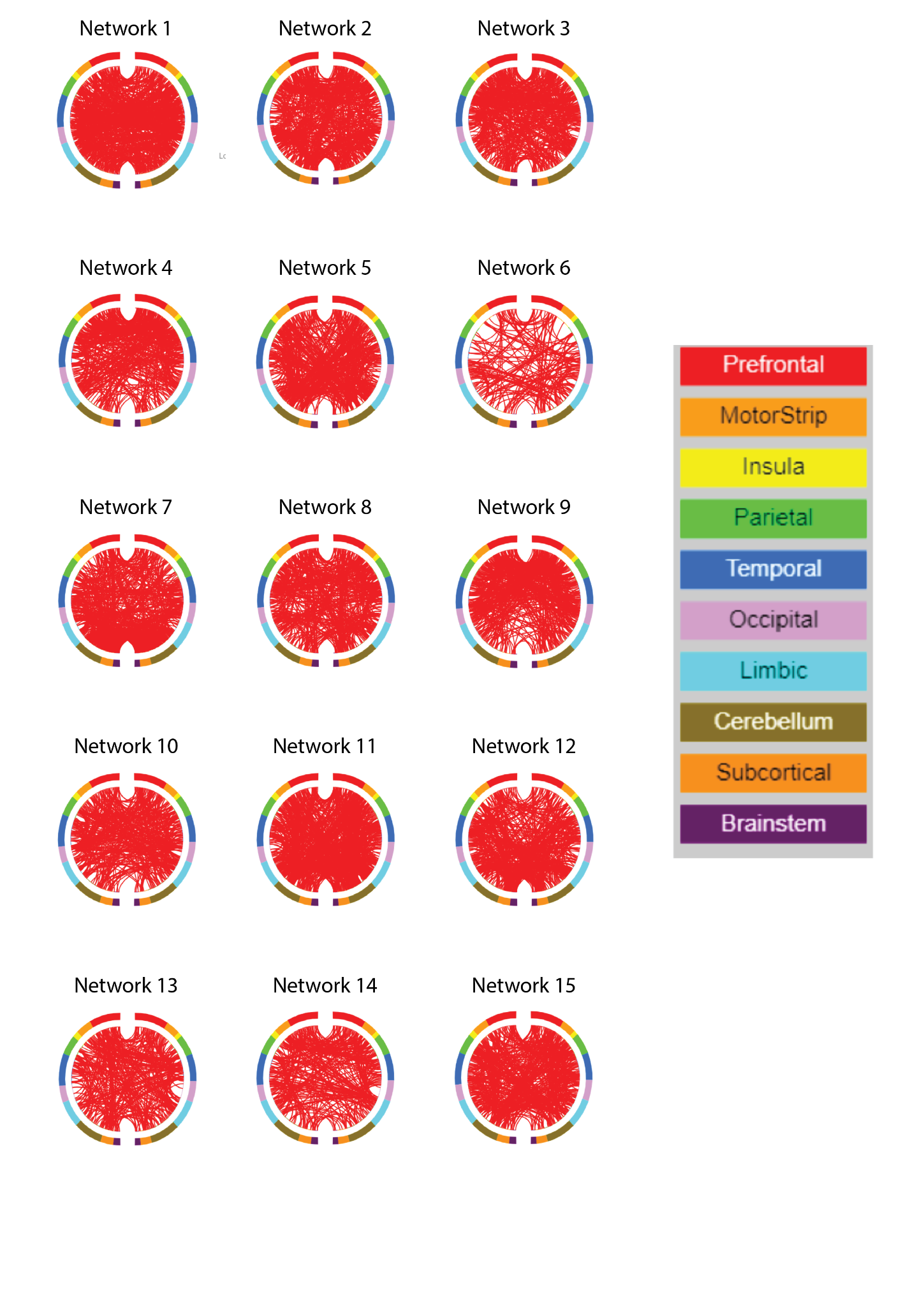


**Figure S14:** Visualization of edge-centric networks 1-15 in the 55 network partition. Each line represents an edge between two nodes which are arranged circularly and further organized by hemisphere and anatomical region. These figures were made with BioImage Suite Web (https://bioimagesuiteweb.github.io/webapp/).


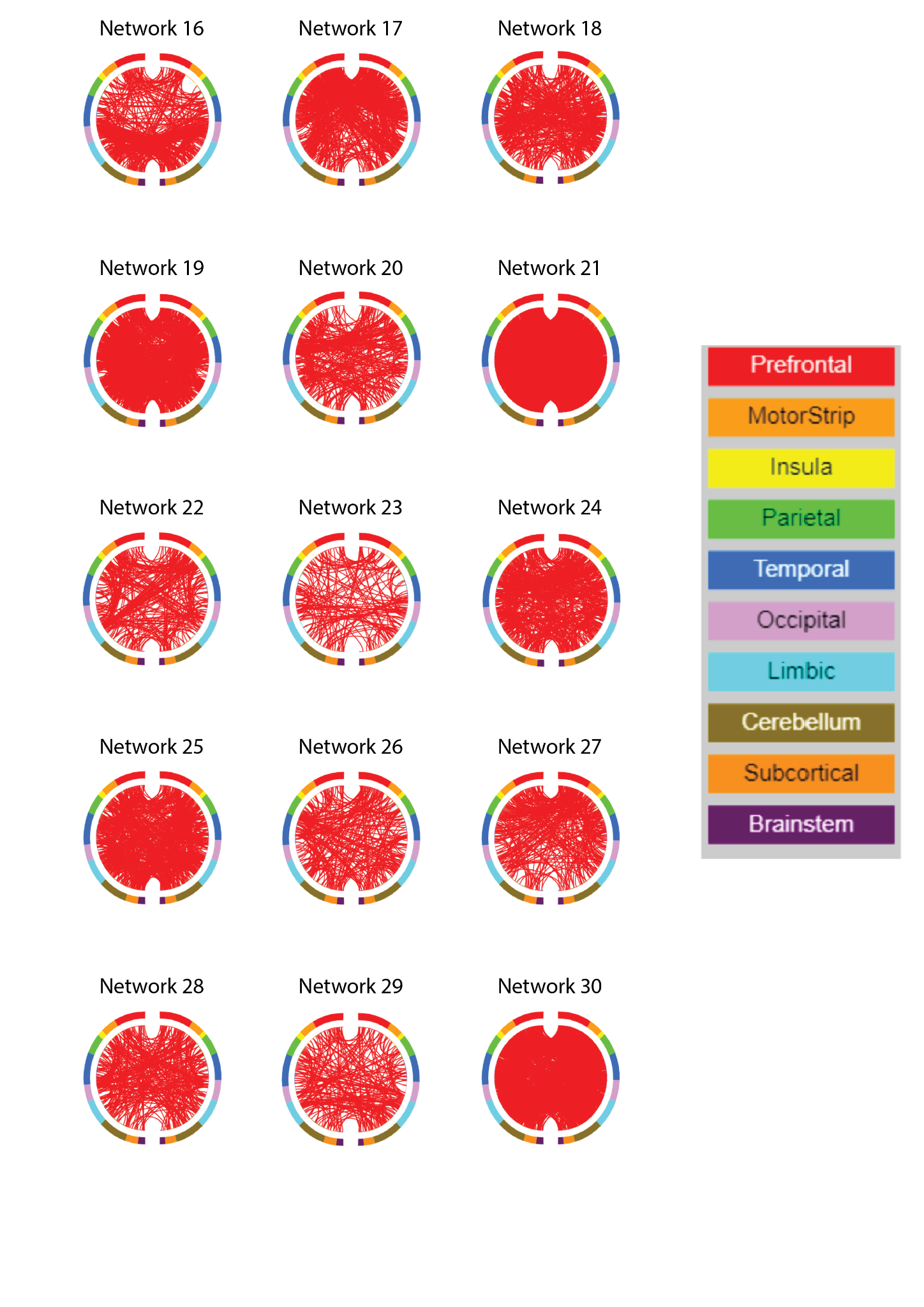


**Figure S15:** Visualization of edge-centric networks 16-30 in the 55 network partition. Each line represents an edge between two nodes which are arranged circularly and further organized by hemisphere and anatomical region. These figures were made with BioImage Suite Web (https://bioimagesuiteweb.github.io/webapp/).


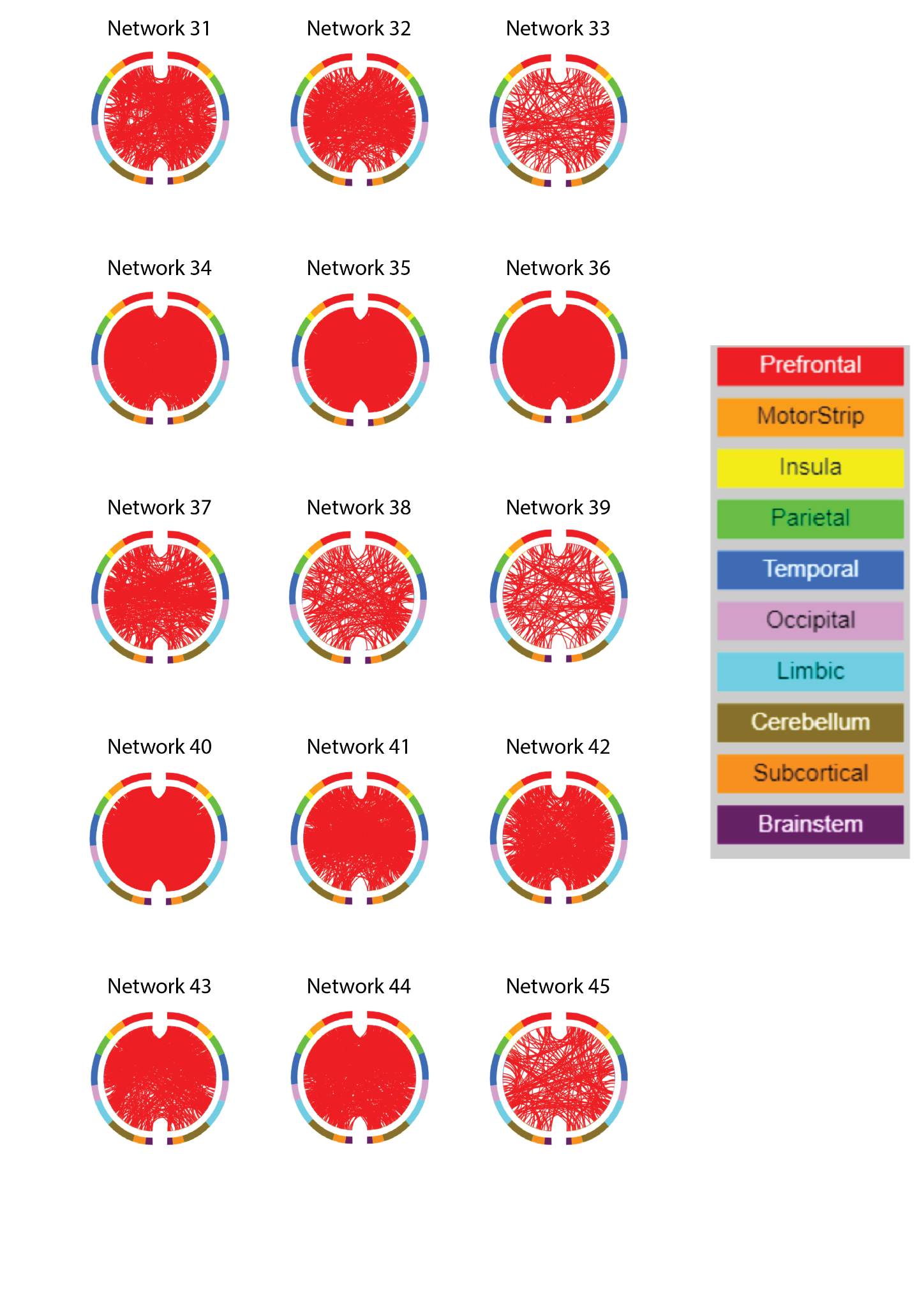


**Figure S16:** Visualization of edge-centric networks 31-45 in the 55 network partition. Each line represents an edge between two nodes which are arranged circularly and further organized by hemisphere and anatomical region. These figures were made with BioImage Suite Web (https://bioimagesuiteweb.github.io/webapp/).


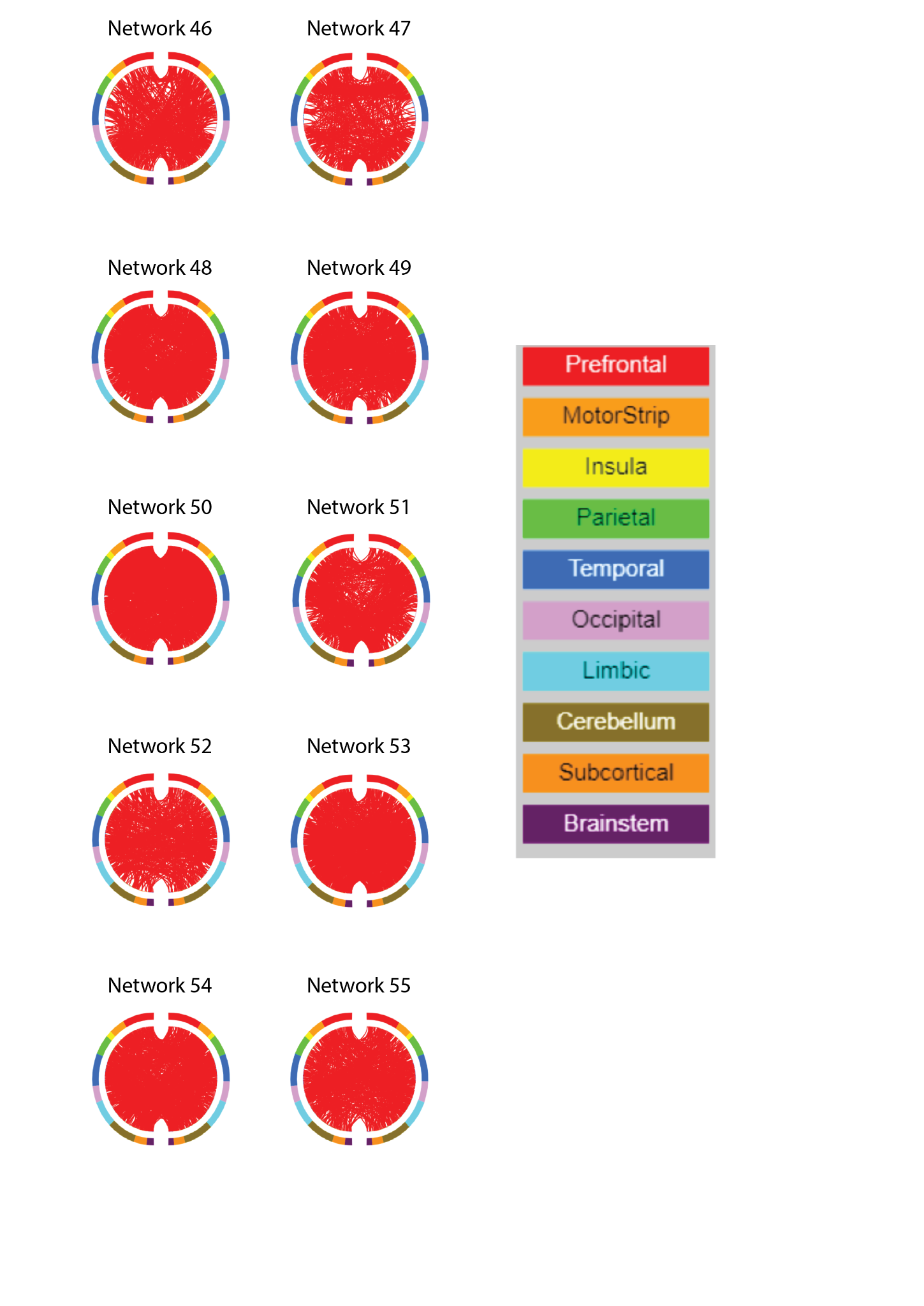


**Figure S17:** Visualization of edge-centric networks 46-55 in the 55 network partition. Each line represents an edge between two nodes which are arranged circularly and further organized by hemisphere and anatomical region. These figures were made with BioImage Suite Web (https://bioimagesuiteweb.github.io/webapp/).
